# Patient-derived organoids recapitulate glioma-intrinsic immune program and progenitor populations of glioblastoma

**DOI:** 10.1093/pnasnexus/pgae051

**Published:** 2024-02-02

**Authors:** Fumihiro Watanabe, Ethan W Hollingsworth, Jenna M Bartley, Lauren Wisehart, Rahil Desai, Annalisa M Hartlaub, Mark E Hester, Paula Schiapparelli, Alfredo Quiñones-Hinojosa, Jaime Imitola

**Affiliations:** Laboratory of Neural Stem Cells and Functional Neurogenetics, Department of Neurology, UConn Health Brain and Spine Institute, 5 Munson Road, Farmington, CT 06030, USA; Departments of Neuroscience, Neurology, Genetics and Genome Sciences, UConn Health, Farmington, CT 06030, USA; Laboratory of Neural Stem Cells and Functional Neurogenetics, Department of Neurology, UConn Health Brain and Spine Institute, 5 Munson Road, Farmington, CT 06030, USA; Departments of Neuroscience, Neurology, Genetics and Genome Sciences, UConn Health, Farmington, CT 06030, USA; Center on Aging UConn Health, Farmington, CT 06030, USA; Laboratory of Neural Stem Cells and Functional Neurogenetics, Department of Neurology, UConn Health Brain and Spine Institute, 5 Munson Road, Farmington, CT 06030, USA; Laboratory of Neural Stem Cells and Functional Neurogenetics, Department of Neurology, UConn Health Brain and Spine Institute, 5 Munson Road, Farmington, CT 06030, USA; The Steve and Cindy Rasmussen Institute for Genomic Medicine, Nationwide Children's Hospital, Columbus, OH 43215, USA; The Steve and Cindy Rasmussen Institute for Genomic Medicine, Nationwide Children's Hospital, Columbus, OH 43215, USA; Department of Pediatrics, The Ohio State University College of Medicine, Columbus, OH 43210, USA; Department of Neuroscience, The Ohio State University Wexner Medical Center, Columbus, OH 43210, USA; Department of Neurosurgery, Brain Tumor Stem Cell Laboratory, Mayo Clinic, Jacksonville, FL 32224, USA; Department of Neurosurgery, Brain Tumor Stem Cell Laboratory, Mayo Clinic, Jacksonville, FL 32224, USA; Laboratory of Neural Stem Cells and Functional Neurogenetics, Department of Neurology, UConn Health Brain and Spine Institute, 5 Munson Road, Farmington, CT 06030, USA; Departments of Neuroscience, Neurology, Genetics and Genome Sciences, UConn Health, Farmington, CT 06030, USA

**Keywords:** organoids, glioblastoma stem cells, interferon, HOPX, SATB2

## Abstract

Glioblastoma multiforme (GBM) is a highly lethal human cancer thought to originate from a self-renewing and therapeutically-resistant population of glioblastoma stem cells (GSCs). The intrinsic mechanisms enacted by GSCs during 3D tumor formation, however, remain unclear, especially in the stages prior to angiogenic/immunological infiltration. In this study, we performed a deep characterization of the genetic, immune, and metabolic profiles of GBM organoids from several patient-derived GSCs (GBMO). Despite being devoid of immune cells, transcriptomic analysis across GBMO revealed a surprising immune-like molecular program, enriched in cytokine, antigen presentation and processing, T-cell receptor inhibitors, and interferon genes. We find two important cell populations thought to drive GBM progression, Special AT-rich sequence-binding protein 2 (SATB2^+^) and homeodomain-only protein homeobox (HOPX^+^) progenitors, contribute to this immune landscape in GBMO and GBM in vivo. These progenitors, but not other cell types in GBMO, are resistant to conventional GBM therapies, temozolomide and irradiation. Our work defines a novel intrinsic immune-like landscape in GBMO driven, in part, by SATB2^+^ and HOPX^+^ progenitors and deepens our understanding of the intrinsic mechanisms utilized by GSCs in early GBM formation.

**Significance Statement**

Glioblastoma is the most lethal brain cancer, and one view is that these tumors originate from malignant glioblastoma stem cells (GSCs). These cells show an increase capacity to generate tumors in vivo; however, how an initial cluster of GSCs forms a 3D tumor is unknown. GSCs are able to form a glioblastoma multiforme (GBM) organoid in vitro, similar to the original tumor; however, how they form, what kinds of cells they originate, the genetic abnormalities, and the metabolism of these organoids are unknown. In this study, we utilize patient-derived GSC and uncover novel cellular and molecular features of GBM organoids that includes the enrichment for an intrinsic glioma stem cells specific immune-like program and STAB2 and HOPX populations of malignant cells harboring an interferon molecular program that may explain how GBM interact with immune cells.

## Introduction

Glioblastoma multiforme (GBM) remains one of the most lethal cancers worldwide (1). One prevailing model of GBM posits that tumors originate and recur from glioblastoma stem cells (GSCs) (1–3). Like other stem cells, GSCs have increased self-renewal capacity and exhibit a progenitor-like state, but differ in their pathological properties, including increased survival, aneuploidy, oncogenesis, and therapeutic resistance (1–3). Beyond the cell-autonomous component of GSCs, GBM evolves by forming a tumor microenvironment (TME). That is, a collection of nontumoral cells, including vascular, immune, and mesenchymal cells, which permit angiogenesis, local immunosuppression (thereby facilitating tumor cell proliferation), migration, and invasion into normal tissue, which altogether leads to explosive tumor growth. This TME crosstalk shapes the intrinsic properties of the developing tumor itself. One important example is that GBM cells regulate infiltrating immune cell types according to their mutational landscape (4) and intrinsic gene expression programs (5). These abilities have implications for the crosstalk between tumor and immune cell compartments that can be exploited therapeutically. Thus, how exactly an initial cluster of GSCs in the preangiogenic/preimmunological infiltration state evolves and co-opt the TME to favor tumor promotion remains a fundamentally unanswered question with important clinical and therapeutic implications.

Preclinical GBM models primarily consist of genetically engineered mouse models or human tumor-derived cell lines, which can be propagated either in 2D cultures or as patient-derived xenografts in mice (6, 7), but there is also growing interest in glioblastoma organoids (8–13). Organoids are defined as 3D in vitro tissue-like constructs derived from isolated stem cells that mimic their corresponding in vivo organ. Among the several models of GBM organoids (8–13), one derived from intact microscopic pieces of tissue from surgically resected tumors is termed GBO. This model offers the advantage of closely replicating its native tumor, as the tissue has not undergone major alterations other than being placed in defined cultures. However, this model is less suitable for studying the early stages of GBM formation, since the resected tissue was already organized in vivo and mixed with infiltrating immune and vascular cells (10). Moreover, by the time GBMs are usually detected, tumor and microenvironment interactions are well established. An alternative GBM organoid model is one derived from GSCs isolated from a patient's resected GBM surgical tissue, which are used to generate GBM organoids that recapitulate early aspects of GBM tissue and organization (11). For this GSC-derived model, the conditions under which GSCs are cultured are critical. For instance, the continuous addition of exogenous growth factors to the organoids not present in the brain may favor clonal selection of certain cells and limit how accurately GSCs recapitulate tumor formation as it occurs in vivo. These features of current GBM organoid models limit our ability to experimentally study the intrinsic properties of human GBM stem cells in a preangiogenic/preimmune infiltration stage.

In this study, we applied a growth factor–free protocol to perform a comprehensive, integrative characterization of immune, genetic, and metabolic phenotypes of GBM organoids generated from several patient-derived GSCs that we term the GBM Stem Cell Modified Organoid Protocol (GBMO). We specifically examined GBMO microanatomy, progenitor diversity, and mutational and transcriptomic landscapes, as they relate to GBM in vivo. We found that GBMO harbor an immune program, driven in part by SATB2^+^ and homeodomain-only protein homeobox (HOPX^+^) progenitors, which we find are uniquely resistant to conventional therapies. Our work advances our understanding of the intrinsic cellular and molecular features of GSC-derived GBMO in the preangiogenic/preimmunological infiltration stages.

## Results

### GBMO recapitulate molecular and cellular hallmarks of GBM in vivo

Before characterizing the intrinsic immune programs of GBMO, we asked if GBMO recapitulated the self-organization, and genomic and metabolic aberrations seen in GBM in vivo. Our results confirmed that indeed, GBMO mimics these hallmark features of GBM (Supplementary Text, Figs. S1–S5, and Tables S2–S6). We thus moved forward in using GBMO as a preangiogenic model of early GBM formation using GSC lines, all of which were functionally validated as bona fide stem cells in vivo and in vitro (Table S1) (14–17).

**Fig. 1.**
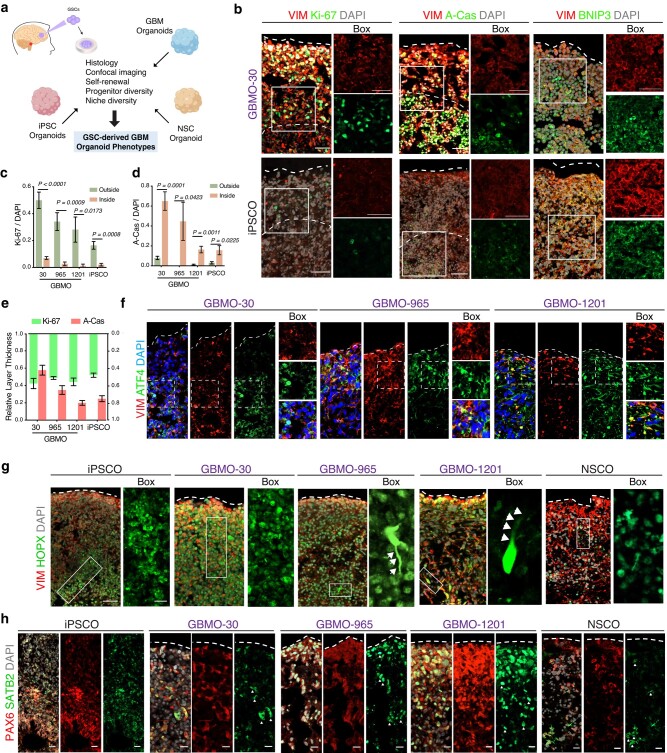
Validation of the GBMO model in the absence of infiltrating immune cells mimicking the progenitor diversity of GBMs. a) Approach for studying patient-derived glioblastoma in the GBM organoid model. b) Representative immunohistochemistry for VIM, proliferative (Ki-67), apoptotic (active-caspase-3 [A-Cas]), and hypoxia (BNIP3) markers in GBMO-30 and iPSCO. Scale bar, 50 μm. c–e) Quantification of Ki-67-positive (c) and A-Cas-positive (d) cells, and relative thickness of proliferative and apoptotic areas (e) in iPSCO and patient-derived GBMO. Bar graphs are presented as means ± SEM. Two-tailed unpaired Student's t test. (Ki-67: GBMO-30, *P* < 0.0001; GBMO-965, *P =* 0.0009; GBMO-1201, *P =* 0.0173; iPSCO, *P =* 0.0008. A-Cas, GBMO-30, *P* = 0.0001; GBMO-965, *P =* 0.0423; GBMO-1201, *P* = 0.0011; iPSCO, *P =* 0.0225.) f) Immunofluorescent images for the quiescent/stress marker ATF4 in GBMO-30, GBMO-965, and GBMO-1201. g) Sample images of immunostaining for VIM, and HOPX markers of oRG in iPSCO, GBMO-30, GBMO-965, GBMO-1201, and NSCO. Scale bar, 20 μm. h) Representative confocal images for immunostaining of deep-layer cortical neuron progenitor (SATB2) and radial glia (PAX6) markers in iPSCO, hNSO, and each GBMO line. Scale bar, 20 μm.

We first investigated progenitor diversity in GBMOs, since in vivo GBMs are considered to harbor unique populations of cancer progenitor cells. We compared GBMO self-organization and progenitor diversity to nontumoral, age-matched cerebral organoids derived from human-induced pluripotent stem cells (iPSCs) (18), which we and others have shown to model the stereotypical architecture of the developing brain (19, 20). GBMO and iPSC organoid (iPSCO), all maintained in Matrigel and growth factor–free culture conditions, uniformly expressed the human radial glial marker, vimentin (VIM) (21). Both organoids were composed of areas of proliferation, marked by Ki-67 (Ki-67^+^), apoptosis by activated caspase-3 (A-Cas^+^), and quiescent/stressed cells by activating transcription factor 4 (ATF4^+^), albeit to a lesser extent in iPSCO (Figs. 1a–f and S6a and b). We quantified Ki-67^+^ and A-Cas^+^ among GBMO and found that GBMO-30 contained marked overlap, while the others exhibited a clear delineation of proliferating cells from the outside surface and apoptotic and quiescent cells from the inner core (Fig. 1c–e). As organoids expand, their inner core experiences a reduction in oxygenation due to diffusion limits (11). To determine hypoxic gradients in GBMO, we stained for the BCL-2-interacting protein-3 (BNIP3), a marker for hypoxia by virtue of its place downstream of hypoxia-inducible factor 1α (22). We found increased cytoplasmic and nuclear BNIP3 expression inside GBMO compared with the surfaces, as shown by quantitative confocal intensity profile measurements (Figs. 1b and S6c–f).

To determine the functional basis for the expression of the radial glia progenitor marker VIM in GBMO, we stained for the phosphorylated form (p-VIM), a marker for radial glia division. Notably, we found a population of p-VIM^+^ radial glia-like tumor cells undergoing mitosis in the proliferating layers of GBMO (Fig. S6g). We confirmed their identity by staining for HOPX, a transcriptional regulator and marker for outer radial glia (oRG) in normal human neurodevelopment. Unlike their stereotypical localization to the outer subventricular zone (SVZ) in iPSCO, we observed HOPX^+^ cells scattered throughout the outer edge of GBMO, some with characteristic polarity and elongated processes (Fig. 1g). This oRG-like population in GBM has been recently described as invasive GBM cells with stem cell and migratory properties in vivo (10, 23, 24). Altogether, these data demonstrate that GBMOs are capable of replicating the pathological oRG-like cell population observed in GBM in vivo and GBM tissue ex vivo (10, 23, 24).

The identification of these oRG-like tumor cells, together with the known ability of GSCs to co-opt developmental programs to direct tumorigenesis (25), prompted us to examine the molecular diversity of progenitor markers in GBMO. Using iPSCO as a control, we observed well-defined SOX2^+^ ventricular zone and SVZ-like areas adjacent to cortical plate-like areas containing CTIP2^+^ cells, a marker for early-born cortical neurons (19) (Fig. S7a). In contrast, we did not observe CTIP2^+^ cells in any GBMO, mirroring its low expression in GBM in vivo. Though unsurprisingly absent in GBM-30, which is characterized by the mesenchymal phenotype in vivo (15, 16, 26), SOX2^+^ cells were variable among patient-derived lines and dispersed throughout the layers of GBMO (Fig. S7a). We stained for additional lineage markers, including PAX6; the intermediate cell marker TBR2; deep-layer neuron markers, SATB2 and TBR1; and Cajal–Retzius cell marker, REELIN (Fig. S7b). Unlike iPSCO, where cells were stereotypically located, these cell-type markers showed disorganized expression scattered throughout the GBMO. Marker expression intensity largely reflected what has been reported in GBM in vivo. For instance, SATB2 (Fig. 1h), a bona fide marker for GSCs that was recently found to be a driver for GBM growth (27), was highly expressed in GBMOs compared with neural stem cell organoid (NSCO), while TBR1 was expressed in all GBMOs (Fig. S7b). Collectively, these results indicate that GSCs form organoids that reactivate a progenitor program like GBM in vivo, including the oRG-like HOPX^+^ and SATB2^+^ cell populations (Fig. S7c and d).

### GBMO-intrinsic gene expression is enriched for unique glia immune-like molecular programs

**Fig. 2.**
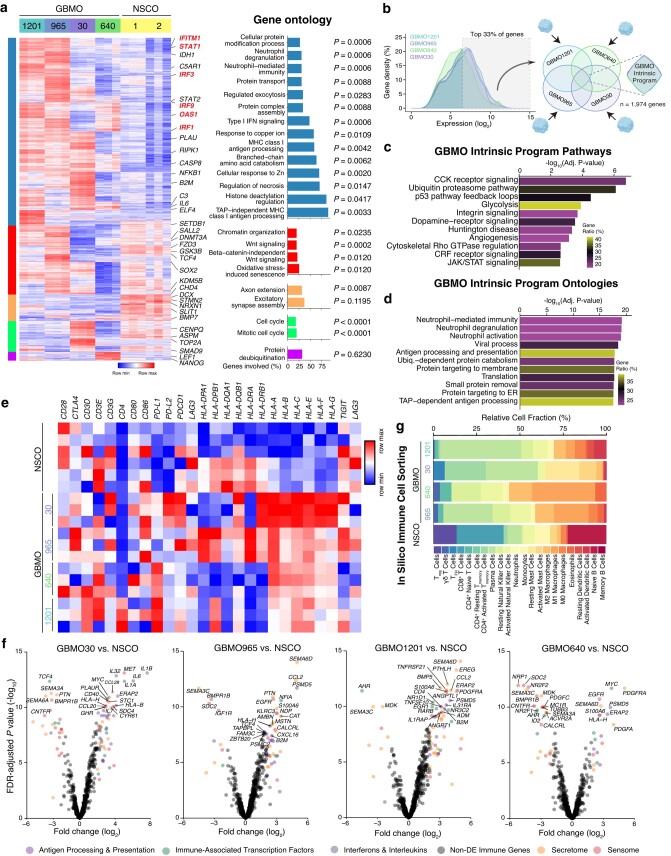
GBMO-intrinsic program analysis reveals enrichment of immune genes. a) Hierarchical clustering heat map of differential gene expression between all GBMO and NSCO with GO for each of the five identified gene clusters. Selected genes from each cluster are highlighted on the right. b) Schematic workflow for identifying genes comprising the GBMO-intrinsic program. Briefly, we focused on the shared top 33% of expressed genes from each GBMO line. c) Pathway enrichment analysis of GBMO-intrinsic program genes. d) GO enrichment analysis of GBMO-intrinsic program genes. e) Heat map for selected transmembrane proteins of immune system in each GBMO and hNSCO. f) Volcano plots of DE of immune genes for each GBMO line, relative to NSCO. Genes are colored by immune category if differentially expressed (logFC > ±2, adj. *P-*value <0.05). g) In silico immune cell transcriptomic analysis for each GBMO line using CIBERSORT to infer immune cell expression signatures within each GBMO line and NSCO.

To understand GBMO gene programs on a global scale, we performed differential expression (DE; defined as log_2_(FC) ± 2 and false discovery rate (FDR)-adjusted *P*-value <0.05) between GBMO and NSCO. We identified 1,743 DE genes (DEGs) across GBMO (Fig. 2a-left and Tables S6–S9). Unsupervised hierarchal clustering of these DEGs yielded five gene clusters of interest (gene ontology [GO], Fig. 2a-right). The largest cluster, containing genes almost exclusively up-regulated in GBMO, was surprisingly enriched for immune signaling genes associated with interferon pathways (*IFITM1*, *STAT1*, *OAS1*, *IRF9*, *IRF3*, *IRF1*, *HLA*, *NFKB1*), cytokines (*IL6*), as well as hallmark processes of cancer (*IDH1*) and (*MEF/ELF4*), a transcription factor associated with stemness in GBM (Fig. 2a, blue columns). The next largest cluster included genes up-regulated in GBMO-1201 and GBMO-965, and NSCO, suggesting both utilize similar chromatin and Wnt signaling pathways (Fig. 2a, red columns). Finally, GBMO-30 was enriched for cell cycle genes, like *ASPM* and *TOP2A*, suggesting it to be the most mitotically active and supporting its highly malignant profile (Fig. 2a, green column).

In contrast, GO and pathway analysis of NSCO-specific clusters indicated enrichment for molecules belonging to human neurodevelopment, consistent with the up-regulation of known neurogenic genes (*DCX*, *STMN2*, and *NRXN1*) in NSCO (Fig. 2a, orange column). We confirmed the diversity of expression of genes proposed to be involved in GBM (25) which showed heterogeneity, but increased expression in all GBMO compared with NSCOs, suggesting that each organoid may have different gene networks that participate in driving their growth (Fig. S8a and b). We confirmed these transcriptomic results by qPCR in GBMO-30 (Fig. S8c).

One advantage of our GBMO system is its lack of stromal cells compared with GBO, which harbors endothelial and immune cells (11). We leveraged this feature to define an intrinsic glial genetic program, modeling a preangiogenic state. To do this, we analyzed the top one-third of expressed genes in each GBMO and focused on the convergence of these genes between all GBMO lines to find a shared molecular program. We found 1,974 genes, which we henceforth refer to as the GBMO-intrinsic program (Fig. 2b). To gain insights into the functional architecture of this program, we performed pathway and ontology analyses. The GBMO-intrinsic program exhibited enrichment for pathways known to be dysregulated in cancer, such as p53, integrin signaling, glycolysis, and angiogenesis (Fig. 2c). GO analysis, on the contrary, highlighted a substantial enrichment for genes involved in immune signaling, consistent with our previous analysis of DEGs (Fig. 2d). Since GBM cells engage in immune interaction with T cells, we next examined the expression of costimulatory/inhibitory pathways in GBMO (28, 29). GBMO showed increased expression of *PD-L1* in GBMO-640 and GBMO-1201, and *PD-L2* in GBM-30. We also observed elevated expression of major histocompatibility complex (MHC) class I genes in all GBMO, especially GBMO-30 (Fig. 2e).

To obtain a more refined view of these immune genes, we evaluated each GBMO compared with NSCO using a collated list of published immune-associated genes (IAGs) from the Immunological Genome Project reference database (30) and separated these genes into functional clusters, representing interleukins (ILs)/interferons (IFNs), sensome, secretome, and immune-associated transcription factors. This clustering yielded insights into the GBMO diversity of immune-like expression (Fig. 2f). For example, most of the up-regulated IAGs in GBMO-1201 belong to secretome and IFN/IL classes, suggesting it may persistently release immune molecules into the TME like *CCL2*, a chemoattract associated with poor prognosis in GBM. GBMO-640 showed expression of *SEMA6D*, *HLA-H*, and *ERAP*2, a protease that functions by trimming antigenic epitopes for presentation by MHC class I molecules. GBMO-30 showed increased *IL6A*, *IL32*, *IL1A*, *ERAP2*, and *HLA-B*. Although there is heterogeneity in immune gene expression, there are some commonly shared molecules among GBMO. We confirm our heterogeneity of gene expression using in silico cell sorting that allocated immune gene expression to all the GBMO (Fig. 2g). To further confirm our results and exclude the possibility that our transcriptome data had been confounded by the presence of a few contaminant immune cells, we stained each GBMO for immune cell markers such as T-cell-specific glycoproteins, CD4 and CD8, and monocyte marker CD68. Indeed, we confirmed their absence in GBMO (Fig. S9a), indicating that these IAGs are intrinsically expressed by GBMO cells.

We next sought to correlate our intrinsic immune GBM organoids findings with GBM in vivo. To do this, we leveraged published single-cell RNA-seq data from GBM and examined the expression of key immune genes that we found up-regulated. Importantly, the analysis of these genes showed that our GBMO-intrinsic program is observed in vivo and is likely not a potential in vitro phenomenon. We analyzed representative genes (*n* = 25) of our immune-like genes in these two GBM datasets and found that in vivo GBM cells intrinsically express these immune molecules. We classified GBM genes with immune function as (i) expressed in tumor compartment only (*intrinsic*); (ii) expressed in both tumor compartment and infiltrating immune cells; or (iii) only in immune cells (*canonical immune genes*), not observed in our GBMO. These data are consistent with the observation that NSCs and GSCs express neuroimmune genes (14, 31–33) (Fig. S9b and c). We finally examined the expression of MHC class I and IFN genes in GBM and found that IFN genes like S*TAT1*, *IRF1*, *TAP1*, *IFITM1*, *IRF3*, and *IRF9* are equally up-regulated in GBM. To confirm the functional *significance* of this gene program, we found that IFN-γ but not IL17 decreased the numbers of GBMO-forming GSC tumorspheres with a concomitant reduction of transcription factor myeloid Elf-1-like factor (MEF), also known as *ELF4* (Fig. S10a–c). Notably, the reduction of *MEF/ELF4* leads to the loss of stemness in GBM (34).

### SATB2^+^ and HOPX^+^ progenitor populations express immune genes in GBM in vivo and GBMO

**Fig. 3.**
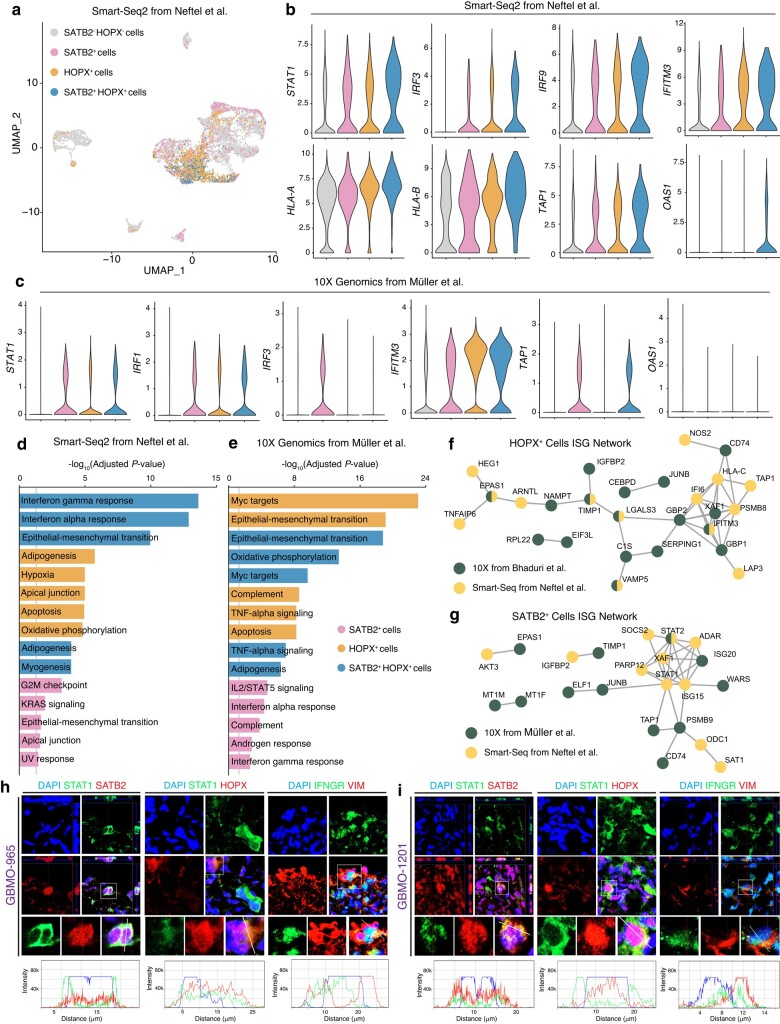
HOPX^+^ and SATB2^+^ cell populations drive immune expression in GBM. a) UMAP plot for in vivo GBM single-cell RNA-seq data from Neftel et al. (35). Individual cells are colored according to their high expression (>50% maximum value) of SATB2 (pink), HOPX (orange), both SATB2 and HOPX (blue), or neither (gray). b) Violin plots for representative IAGs for each cell population from Neftel et al. c) Violin plots for representative IAGs for each cell population from an independent dataset, Muller et al., in which resected GBM were processed for single-cell RNA-seq using 10 × Genomics. d and e) Pathway enrichment analysis for genes up-regulated in SATB2^+^, HOPX^+^, SATB2^+^HOPX^+^ cells in Neftel et al. (d) or Muller et al. (e). Gray line denotes 5% level of significance threshold for log-adjusted *P*-value scores. f and g) Protein–protein interaction network of up-regulated IFN-stimulated genes in SATB2^+^ and HOPX^+^ cells. Nodes are colored by which dataset they were found to be up-regulated. Green, Muller et al.; Yellow, Neftel et al. h and i) Confirmation of expression of IFN-related genes on SATB2 and HOPX cancer progenitors in two independent GBMO. 3D-reconstructed *z*-stacked representative confocal images for immunostaining of STAT1 and IFNGR in HOPX and SATB2 populations in GBMO 1,201 and 965. Scale bar, 20 μm.

Next, we sought to determine which cells were driving the expression of immune genes in GBMO compared with GBM (Fig. 3a). Given that oRG expresses STAT transcription factors and functional IFN receptors (36, 37), we hypothesized that this population, along with SATB2^+^ cells, which are preferentially born from HOPX^+^ oRG during normal neurogenesis, may contribute to the immune expression of GBMO. To address this, we first analyzed cells that highly expressed SATB2, HOPX, or both together in the datasets and compared the expression of critical interferon-stimulated genes (ISGs) among SATB2^+^, HOPX^+^, SATB2^+^HOPX^+^, and HOPX^−^SATB2^−^ cells (Fig. 3a). Strikingly, we found elevated levels of several immune genes, including *STAT1*, *IRF3*, *IRF9*, *IFITM3*, *HLA-A*, and *TAP1* among others in the SATB2^+^HOPX^+^ cells (Fig. 3b). For rigor, we repeated this analysis on a separate single-cell GBM dataset and found similar results (Fig. 3c). To unbiasedly examine the gene signatures of this cell population, we performed DE between the groups and focused on the most up-regulated genes in these cell populations. Pathway enrichment analysis in both datasets revealed these populations shared an enrichment for genes related to epithelial mesenchymal transition, IFN-γ, and IFN-α signaling (Fig. 3d and e). To gain insights to the active immune-related networks of SATB2^+^ and HOPX^+^ cells, we then performed protein–protein interaction analysis, focusing on the topmost up-regulated IFN-stimulated genes (Fig. 3f and g). While these two populations shared some proteins like *TAP1*, *XAF1*, and *CD74*, there were also some cell-type-specific proteins, including *HLA-C* and *IFITM3* in HOPX^+^ cells and *STAT1* and *STAT2* in SATB2^+^ cells. Finally, we confirmed the in vivo GBM and GBMO mRNA findings, at the protein level by performing an analysis on the expression of IFN-γ receptors and STAT1 in SATB2^+^ and HOPX^+^ cells in our GBMO. Immunostaining revealed that *STAT1* and *IFNGR1* were indeed expressed by these cells in GBMO-965 and GBMO-1201, indicating that our GBMO recapitulates part of the immune gene landscape of HOPX^+^ and SATB2^+^ populations from GBM in vivo (Fig. 3h and i).

### Temozolomide and irradiation target GBMO, but spare HOPX^+^ and SATB2^+^ cells

**Fig. 4.**
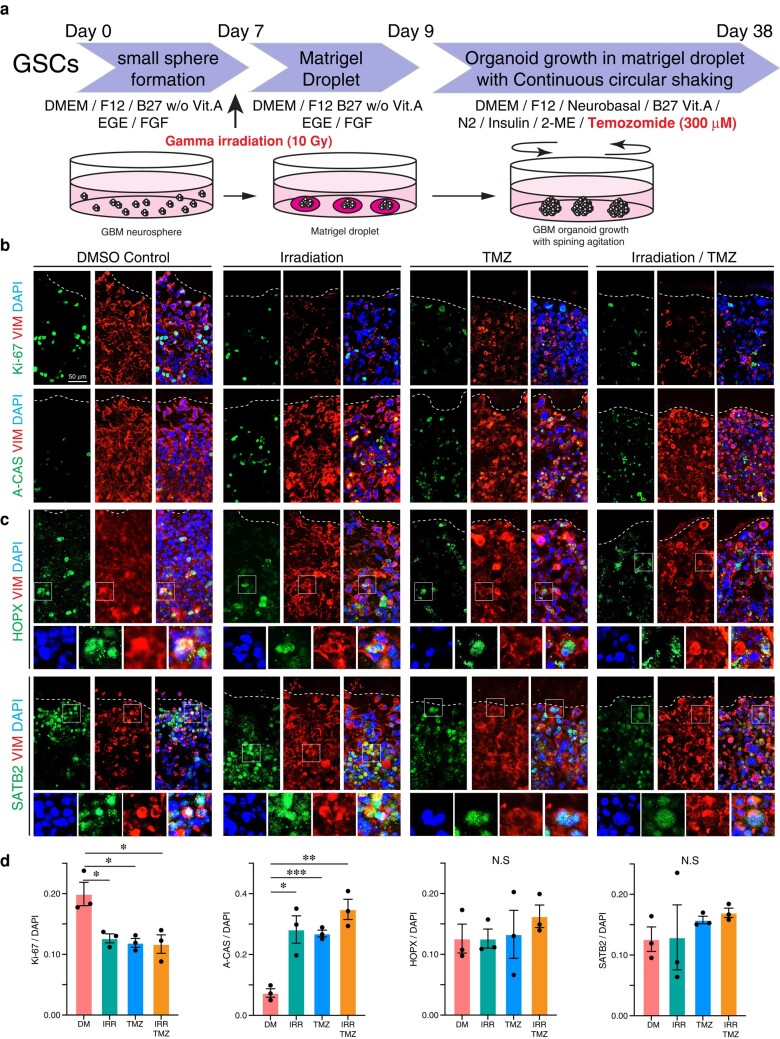
The effects of TMZ or irradiation in GBMO generation. a) Diagram for GBM organoid generation and treatment with chemotherapeutic agent (TMZ) and gamma irradiation (10 Gray: Gy). b) Immunofluorescent images for proliferation (Ki-67, upper) and apoptosis (A-CAS, lower) in GBMO-30 with DMSO control (DM), irradiation (IRR), TMZ and IRR/TMZ combination (IRR/TMZ). c) Immunostaining for radial glia (HOPX, upper) and neural differentiation (SATB2, lower) in GBMO-30 with DM, IRR, TMZ and IRR/TMZ. d) Quantitative analysis for Ki-67, A-CAS, HOPX, and SATB2-positive cells in GBMO-30 with DM, IRR, TMZ, and IRR/TMZ. One-way ANOVA with Bartlett's test; *n* = 3 independent experiments. Data presented as mean ± SEM (Ki-67: DM vs. IRR, *P* = 0.0238; DM vs. TMZ, *P* = 0.0171; DM vs. IRR/TMZ, *P* = 0.0279. A-CAS: DM vs. IRR, *P* = 0.0115; DM vs. TMZ, *P* = 0.0004; DM vs. IRR/TMZ, *P* = 0.0016).

One critical question is whether GBMOs are sensitive to the current treatments of GBM, irradiation and/or temozolomide (TMZ). To test this, we exposed GBMO-30, GBMO-965, and GBMO-1201 to irradiation or TMZ. All GBMO showed a decrease in the number of Ki67^+^ proliferating cells and a decrease in Cas3^+^ apoptotic cells (Figs. 4 and S11). Intriguingly, when we co-stained with HOPX and SATB2, we found the proliferation and apoptosis of these cells were unaffected by treatment with irradiation or TMZ. To test whether the combination of TMZ and irradiation could overcome this resistance, we concomitantly treated GBMO30, the most malignant of our GBMOs, with TMZ and irradiation. Still, however, the population of HOPX and SATB2 progenitors remained unchanged from GBMO controls (Figs. 4 and S11).

## Discussion

**Fig. 5.**
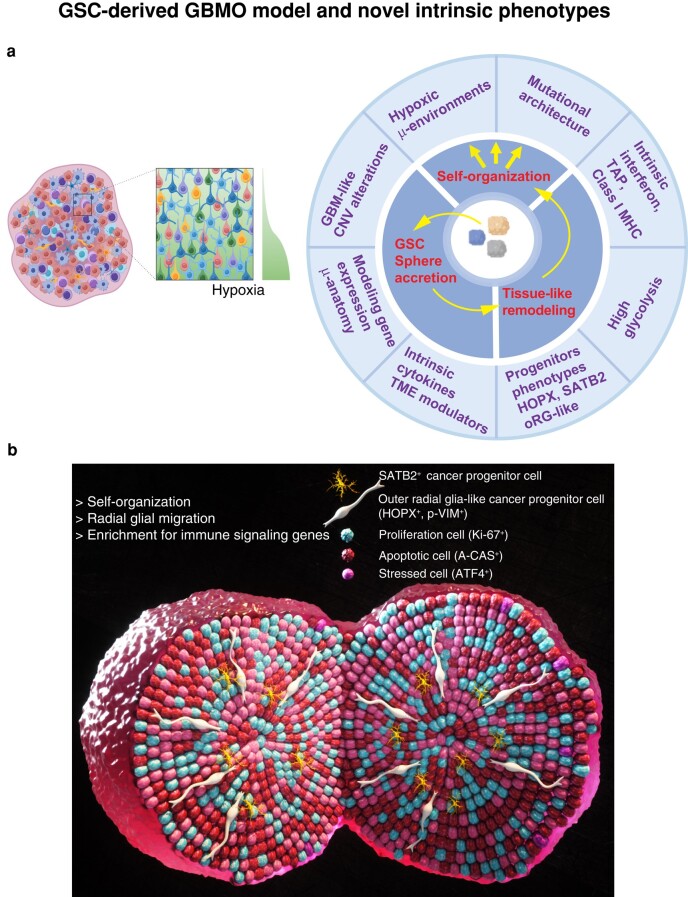
GBM organoid model and novel intrinsic phenotypes. a) Schematic of the GBMO organoid model depicting observed novel phenotypes and microanatomy of GBMO resembling GBMs in vivo. b) 3D model of a GSC-GBMO organoid summarizing our novel findings, including SATB2 and HOPX oRG-like progenitors that are characterized as bona fide glioma stem cells. This 3D model shows diverse microanatomical domains and hypoxic microenvironments, intrinsic immune program enriched with MHC class I, IFN, and inhibitory pathways in SATB2 and HOPX oRG cells.

Our understanding of the early molecular programs of GBM tumor formation by GSCs remains limited by a scarcity of models. Typically, studying GSCs is done by injecting these cells into mouse brains, as a xenotransplant into an immunosuppressed host. However, because mice are immunosuppressed, this approach is less suitable to dissect intrinsic neuroimmune programs and interactions (38) seen in neuroinflammation (1, 39) and brain cancers (3). In this study, we performed a detailed characterization of the intrinsic properties of patient-derived GBMO from GSCs to significantly expand our understanding of the remarkable ability of GSCs to form a 3D tumor environment from a small, highly malignant group of stem cells (Fig. 5). By defining the intrinsic programs of GBMO in the absence of immune and vascular contamination, we found an unexpected immune-like program enriched in GSC-derived cell populations that are patient specific.

In characterizing GBMO, we found the in vitro recapitulation of oRG-like cells that have been recently identified in GBM in vivo (23). These oRG-like cells were present in all patient-derived GBMOs, suggesting they may be ubiquitous in GBM. Underscoring their importance is the hypothesis that human GBM initiation originates with aberrant reactivation of the oRG genetic program (36). In our GBMO, these oRG-like tumor cells showed migratory phenotypes and localized to the leading edge. It has also been recognized that a subpopulation of oRG may contain an intrinsic mesenchymal population (40), perhaps explaining their ability to express immune genes in GBM. Our GBMO also recapitulated the presence of SATB2^+^ tumor cells known to drive GBM pathology. In normal human brain development, oRG preferentially differentiates into SATB2 upper-layer neurons. Thus, it is tempting to speculate that SATB2 expression may simply be gene-activated downstream of the aberrant deployment of an HOPX oRG-like program. These observations add to the growing evidence that GBM growth and invasiveness may rely on malignant hijacking of an oRG-like gene program.

Our immune findings restructure our view of the immune microenvironment in GBM, which is traditionally seen as driven by infiltrating immune cells, such as macrophages and T cells, rather than GBM cells per se. Instead, they suggest a model in which GBM cells already harbor a patient-specific intrinsic immune program that influences TME formation long before massive vascularization occurs when major classical immune infiltration happens. It may be possible to tailor immunotherapies to the predominant immune repertoire of these cancer cells (4), though such heterogeneity will first need to be confirmed in vivo. We identified novel tumor-autonomous immune programs in GBMO, including strong expression of HLA, tapascin, and IFN genes. Using single-cell RNA-seq and immunostaining, we localized some of this immune expression to oRG-like HOPX^+^ and SATB2^+^ cells. We demonstrated the functional role of this pathway and showed that IFN-γ, a cytokine produced by CD8^+^ T cells, can target GBMO, causing a down-regulation of molecular pathways involved in stemness. In fact, this is supported by recent data from a clinical trial that suggests reverting the T-cell exhaustion in GBM by anti-PD1 immunotherapy leads to a restoration of IFN-γ-producing T cells and improves survival due to T cells targeting the tumor via IFN-γ (41). Whether such benefit derives from preferential targeting of the highly invasive and immunogenic HOPX^+^ and SATB2^+^ cell populations warrants future detailed investigation. Likewise, it will be important to determine whether HOPX and SATB2 progenitors play a causative role in the immune landscape of GBMO, as our results are all correlative.

There are several models of GBM organoids, each with different derivations and nomenclature. These include (i) genetic activation of oncogenes in normal organoids (8, 13); (ii) invasion of normal iPSC-derived brain organoids by GSCs (9, 12); (iii) GBM organoids derived from fresh pieces of GBM, which include stromal vessels and immune cells (GBO) (10); (iv) GSC-derived GBM organoids plus Epidermal Growth Factor/Fibroblast Growth Factor (EGF/FGF) (11); and (v) our feeder-free GBMO system. These models each have their own unique advantages and limitations. We suggest, for instance, that GSC-derived models may be best suited to model the prevascular stages of GBM before initial interactions between the tumor and the TME. Despite the insights into immune biology we found in our GSC-derived GBMO, future studies will also benefit from incorporating GBMO with stromal, vascular, and infiltrating immune cells. Alternatively, orthotopic implantation of GBMO in mice would enable integration with the vasculature in an in vivo environment but with the shortcomings of nonautologous immune cells. Future approaches for vascularization could implement microfluidics on-a-chip with human vascular and immune cells to expand our preangiogenic model to a more in vivo–like model that shares the same immune repertoire for future immunotherapeutic target identification (Fig. S11). Future studies will need to directly compare which model is the most translationally relevant. In addition, whether differences exist in the biology of GBMO seeded from initial, naive GSCs, as modeled here, vs. recurrent, therapy-resistant GSCs is likewise an important question. Finally, comparing the IDH mutant with the wild type represents another pressing question. Of note, we attempted to generate GBMO using IDH-mutant GSCs using our current protocol, but the GSCs were unable to grow (data not shown). Thus, new GBM organoid protocols are needed to accommodate this important subtype.

In conclusion, we demonstrate that GSC-derived GBMO not only models early features of GBM formation but also enables identification of intrinsic molecular programs, including a hitherto unreported immune landscape. Our GBMO model comprises an important addition to preexisting models specialized for understanding both basic GBM biology and patient-specific intrinsic immune vulnerabilities. Much like creating a patient's tumor avatar, GSC-derived GBMOs provide an opportunity for preclinical interrogation of patient-derived cells toward personalizing treatments or immunotherapies, which currently have a median survival of only 18 months (14).

## Methods

### Generation of organoids and cell culture

Human GBM stem cells (GSCs) were obtained from patients and allocated for human research purposes, per the protocols approved by the Institutional Review Board (IRB) at the Mayo Clinic (IRB:16-008485), all participants provided informed consent at the Mayo clinic prior to surgery and procurement of the research samples, which were then used as deidentified cell lines according to OSU and UConnHealth regulations. Confirmatory assays of GSCs for these lines have been demonstrated elsewhere (Table S1). All GSCs used in the study were authenticated and were free of *Mycoplasm* sp. (Fig. S1). Patient-derived GSCs were isolated and cultured as described in Supplementary Methods. All cell lines were handled in accordance with the IBC biosafety practices and relevant ethical guidelines of The Ohio State University College of Medicine, Nationwide Children's Hospital, and University of Connecticut that regulate the use of human cells for research.

### Histology and immunofluorescence

Tissues were fixed in 4% paraformaldehyde for 20 min at 4 °C followed by washing in PBS three times for 10 min. Tissues were allowed to sink in 30% sucrose overnight, embedded in OCT compound (Tissue-Tek, Sakura Finetek USA, Torrance, CA, USA), and then cryosectioned at 20 μm. Tissue sections were stained with hematoxylin and eosin, and images were taken with a light microscope (BX41, Olympus, Tokyo, Japan) equipped with a digital camera (DP71, Olympus). Immunofluorescence and quantitative analysis were performed as described in Supplementary Methods. Images were taken with a confocal laser-scanning microscope (LSM800, Carl Zeiss Microscopy GmbH, Jena, Germany).

### Targeted parallel sequencing and copy-number variation analysis

A custom capture-based, targeted next-generation sequencing panel, which includes probes covering the coding sequences of 407 cancer-related genes and genome-wide copy-number variation (CNV) of backbone targets (Agilent OneSeq 300 kb CNV Backbone + custom panel), was utilized in this study. Sequencing libraries and analysis were done as described in Supplementary Methods.

### Real-time qPCR, transcriptome analysis, and DE

RNA was extracted from cell cultures with QIAzol reagent and miRNeasy Mini Kit (QIAGEN, GmbH, Hilden, Germany), following the manufacturer's protocol. cDNA was obtained from 500 ng of mRNA using the retrotranscription kit (Thermo Fisher Scientific). Real-time qPCR was performed as described in Supplementary Methods. All primer sequences can be found in Table S14. The GeneChip Human Transcriptome Array 1.0 (also known as Clariom D assays; Affymetrix, Thermo Fisher Scientific Inc.) was used to provide a detailed analysis of the organoid transcriptome analysis and was performed as described in Supplementary Methods.

### Principal component and subtype analysis, hierarchal clustering, and functional annotation

A detailed principal component analysis is described in Supplementary Methods.

### Unbiased search strategy for GSC molecular vulnerabilities

To establish an unbiased GBMO-intrinsic genetic program, normalized intensity values for each GBMO line were first filtered to include only the top one-third of highly expressed genes. The resulting gene lists were compared among GBMO lines, and the genes shared by all four lines were the only ones further considered. Gene lists were inputted into Enrichr for enrichment analyses (42). To further ascertain the immune expression states of each GBMO line, we concentrated on the expression of IAGs, as obtained from ImmPort (http://www.immport.org/immport-open/public/home/home) and InnateDB (http://www.innatedb.com) (43), using the original DE analyses to ensure all IAGs were encompassed. Plots were produced using ggplot2 package in R. Again, the convergence of the DE IAGs was utilized and then filtered to focus only on known human transcription factors, as specified by http://fiserlab.org/tf2dna_db//index.html. For heat map generation, data were imported into the online matrix software, Morpheus (https://software.broadinstitute.org/morpheus).

### Processing of single-cell RNA-seq datasets from GBM in vivo

Raw read count matrices for Neftel et al. (35) and Muller et al. (https://www.biorxiv.org/content/10.1101/377606v1.full (44)) were downloaded from GSE131928 and UCSC Single Cell Browser (https://cells.ucsc.edu/), respectively. A Seurat object was created for each matrix separately, and datasets were scaled. The expression of *HOPX* and *SATB2* was then visualized. In each dataset, an expression cutoff of 50% of maximal gene expression was established and all cells expressing at or above this were deemed to be HOPX^+^ or SATB2^+^. Since we found the expression of these genes was not mutually exclusive, we created a third category, HOPX^+^SATB2^+^, for cells expressing both of these genes. Cell populations were identified according to these population criteria, and the expression of well-known IAGs was visualized. Up-regulated genes in each cell population were then determined for each population using the FindAllMarkers command of Seurat v3 (45). From these up-regulated gene lists, pathway analysis was performed using Enrichr. After noting an enrichment of immune-related genes across both datasets, we extracted IFN-stimulated genes from the up-regulated IFN-stimulated genes. We then made use of StringDB (https://string-db.org/) to generate protein–protein interaction networks for the top 25 up-regulated ISGs of each cell population and merged these networks from each dataset together.

### Retrospective analysis of gene expression in human gliomas

Gene expression of neurodevelopmental progenitor markers and up-regulated GBMO genes was determined across primary patient gliomas and subtypes of GBM tumors, determined through analysis of the National Cancer Institute Repository for Molecular Brain Neoplasia Data (http://betastasis.com/glioma/rembrandt/) and TCGA (https://tcga-data.nci.nih.gov/publications/tcga), respectively. Gene expression localization in the structures of primary patient GBM was determined through analysis of the Allen Institute of Ivy GAP (http://glioblastoma.alleninstitute.org/). Expression data were downloaded, and heat maps were generated using the matrix visualization software, Morpheus. Heat maps of Pearson similarity matrices were also generated using Morpheus. Detailed information on heat maps, including gene names in retained order as in Fig. S5, can be found in Tables S10–S14.

### Metabolic analysis of GBMO with Seahorse technology

For Seahorse Analysis (XFe96, Agilent Technologies), organoids were first dissociated via dissociation reagent. Dissociated cells were washed into warmed Seahorse XF DMEM medium supplemented with 10 mM glucose, 1 mM pyruvate, and 2 mM glutamine and plated at a density of 1 × 10^5^ cells/well on a poly-l-lysine-coated XFe96 Seahorse cell culture microplate. Cells were simultaneously tested for oxygen consumption rate and extracellular acidification rate per the manufacturer's XF Real-Time ATP Rate Assay Kit protocol. Mitochondrial ATP and glycolytic ATP production rates were calculated via Agilent Seahorse XF Real-Time ATP Rate Assay Report Generator. ATP production rates were analyzed via t test or ANOVA with Bonferroni post hoc corrections as needed, with significance set at *P* < 0.05.

### Statistical analysis

All the data were included for statistical analyses using GraphPad Prism 6.0. Unpaired Student's t test (two-tailed) was used for the comparison between two unpaired groups, and one-way ANOVA was applied for multigroup data comparisons. The variance was similar between the groups that were being statistically compared. All data met the assumptions of the tests. Survival estimates were calculated using the Kaplan–Meier analysis. Briefly, the expression levels of target genes and patient survival information from the TCGA database were loaded into X-Tile as a tab-delimited text file. By running the “Kaplan–Meier” program, the cohort was then divided into two datasets with the optimal cut points generated according to the highest w2-value defined by log-rank test and Kaplan–Meier analyses. Bar graphs were presented as means ± SEM with statistical significance at *P < 0.05, **P < 0.01, or ***P < 0.001.

## Supplementary Material

pgae051_Supplementary_Data

## Data Availability

All data are included in the manuscript and Supplementary material.

## Preprints

This article was posted on a preprint server: https://doi.org/10.1101/2021.10.06.463228.
