# Supplementary material for: Patient-derived organoids recapitulate glioma-intrinsic immune program and progenitor populations of glioblastoma
Source: PNAS Nexus. 2024 Feb 2;3(2):pgae051. doi: 10.1093/pnasnexus/pgae051 (PMC10879747; doi:10.1093/pnasnexus/pgae051)
Supplement: pgae051_Supplementary_Data [file pgae051_supplementary_data.zip › PNASNEXUS-PNASNEXUS-2022-00087R-s02.pdf]

Species-specific PCR Evaluation

Species	GBM-30	GBM-965	GBM-1201	GBM-640
mouse	-	-	-	-
rat	-	-	-	-
human	+	+	+	+
Chinese hamster	-	-	-	-
African green monkey	-	-	-	-

Marker Analysis

Marker Name	GBM-30	GBM-965	GBM-1201	GBM-640
AMEL	X	X	X, Y	X
CSF1PO	12, 13	11	10	10, 12
D13S317	12	11, 12	11	8, 12
D16S539	9	9, 13	11, 12	9, 11
D5S818	12, 13	11	7, 12	11, 12
D7S820	10	10, 11	11	10
TH01	9	9, 10	7, 8	6, 9.3
TPOX	9	8	8, 11	8
vWA	15	19	17, 19	15

PCR EVALUATION

Marker Name	GBM-30	GBM-965	GBM-1201	GBM-640
Mycoplasma sp.	-	-	-	-

Legend: + = positive - = negative id:id = pooled sample range
id+id+id = non-range pooled sample NT or blank = no test performed
sus = suspect wps = weak positive XX = Testing in progress

Supplementary Figure 1

Comparison of GBM tissue and Organoids from the same patient

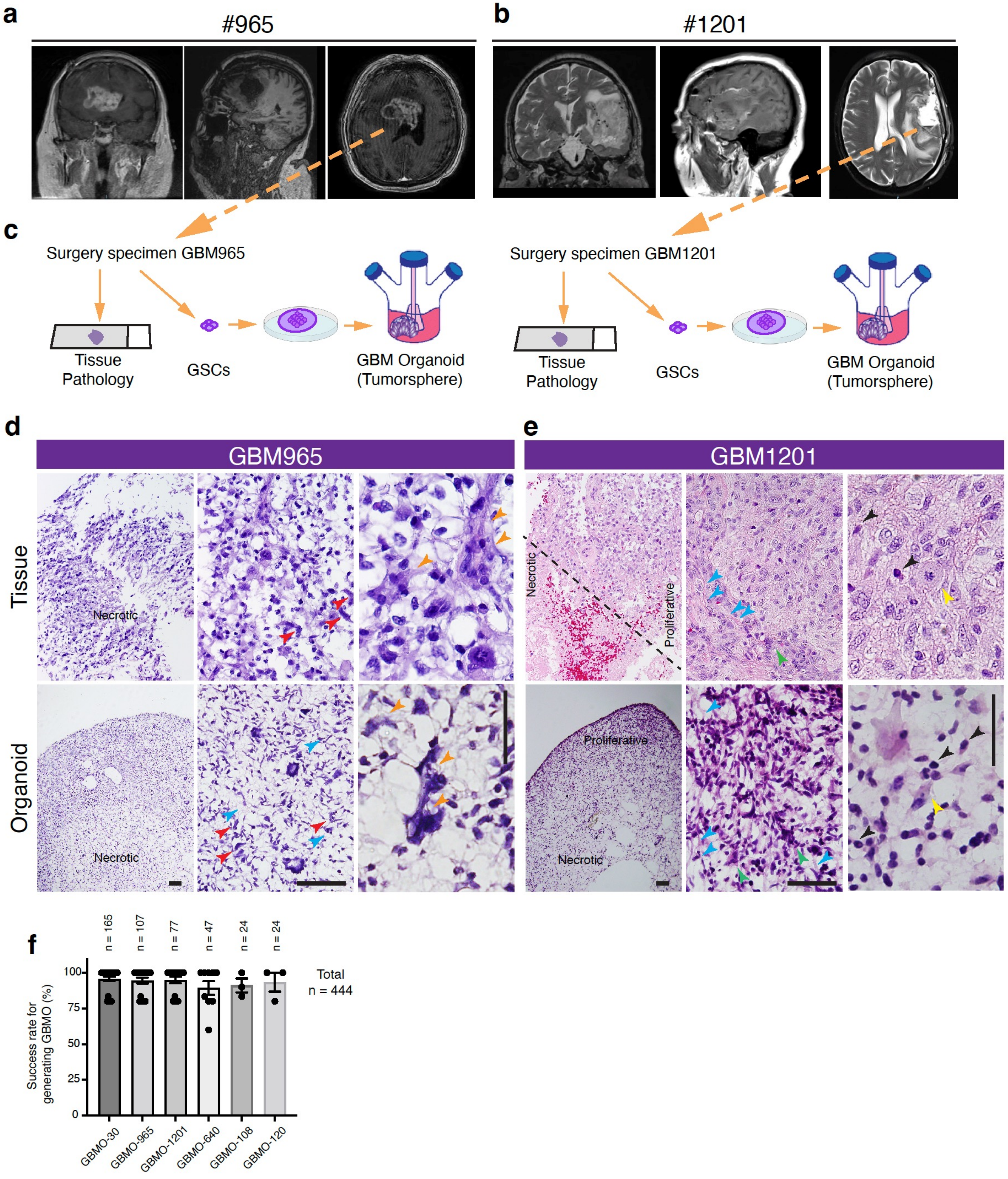

Supplementary Figure 2

a

Comparison of properties GBM neurospheres vs GBMOs

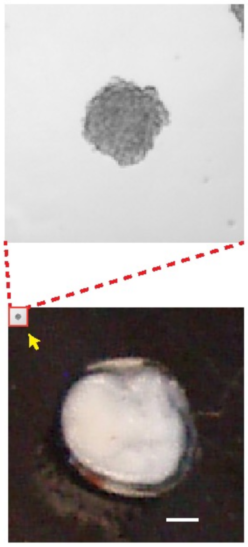

Feature	GBM Neurospheres (GSCs)	GBM Organoid (GBMO)
Size	Diameter: 200 μm	Diameter: 4000 μm (20 Fold)
Layering Organization	Layers Absent Lacks Self-Organization	Layers Present Tissue-Like Self-Organization
Niches	Niches Absent	Apoptotic, Proliferative, & Hypoxic Niches
Self-Renewal	Yes, not after differentiation	Yes, always retained
Transcriptome	N.D.	In Vivo-like Recapitulation

b

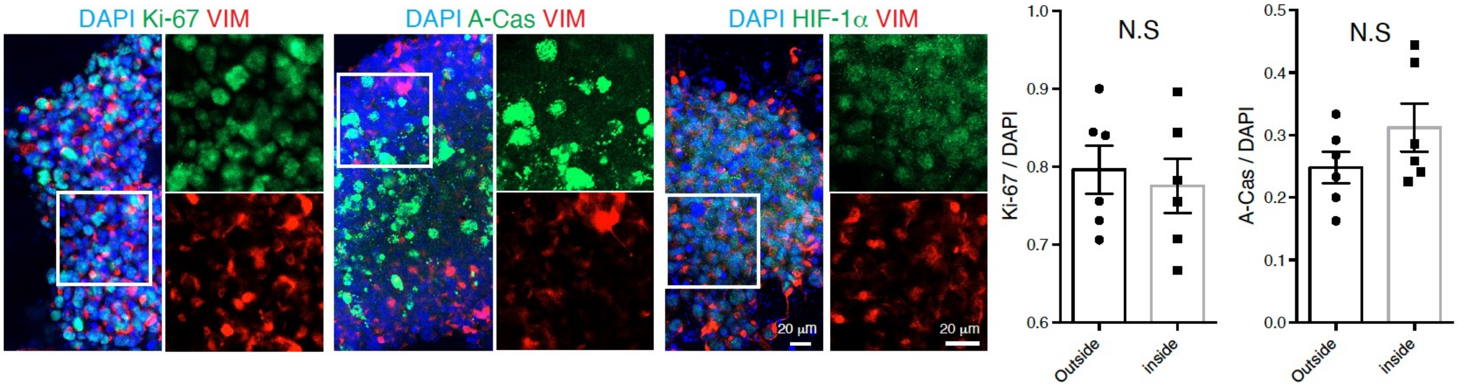

Supplementary Figure 3

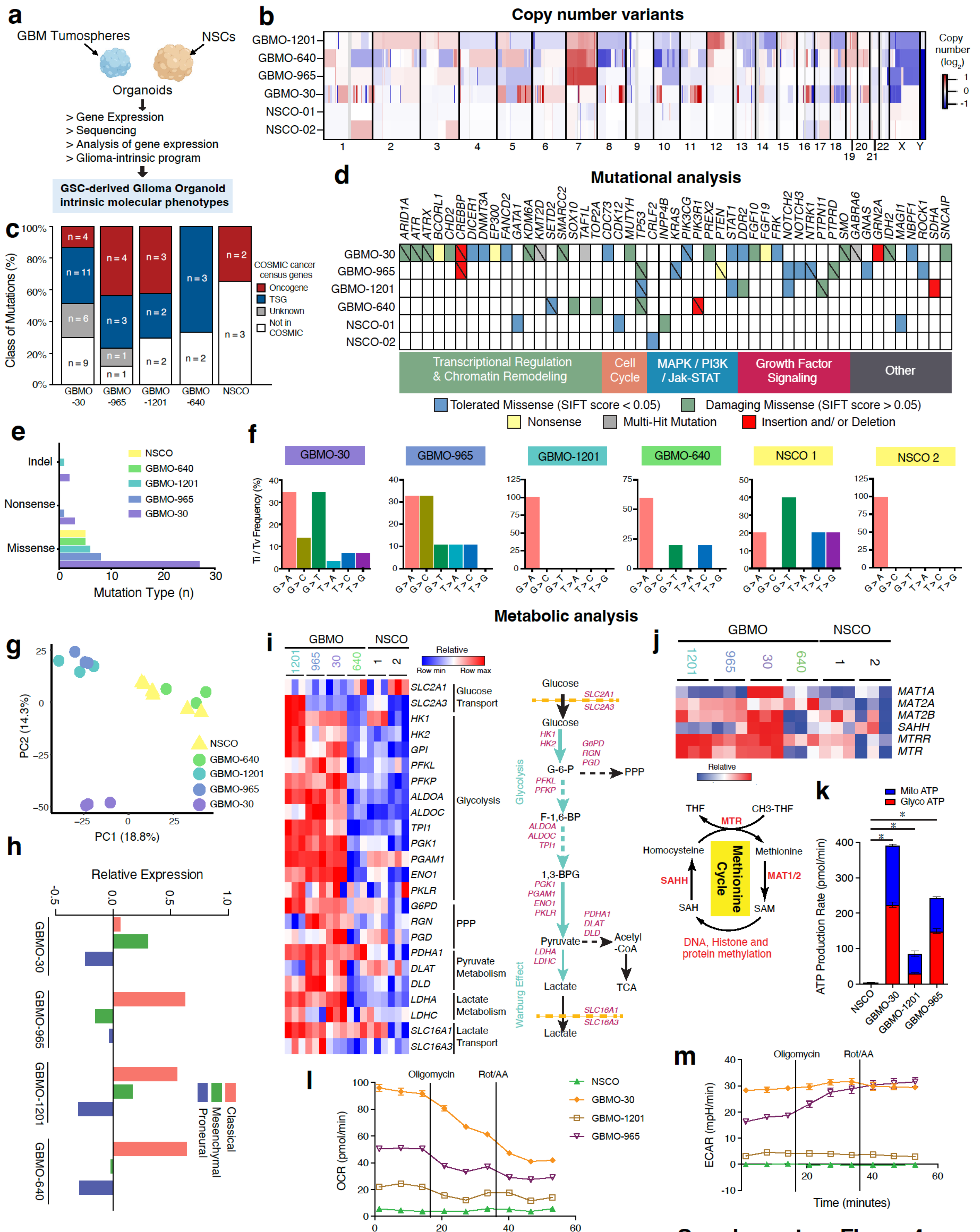

Supplementary Figure 4

Correlation of differential expression of GBMO genes in in vivo GBM microanatomy by IvyGAP

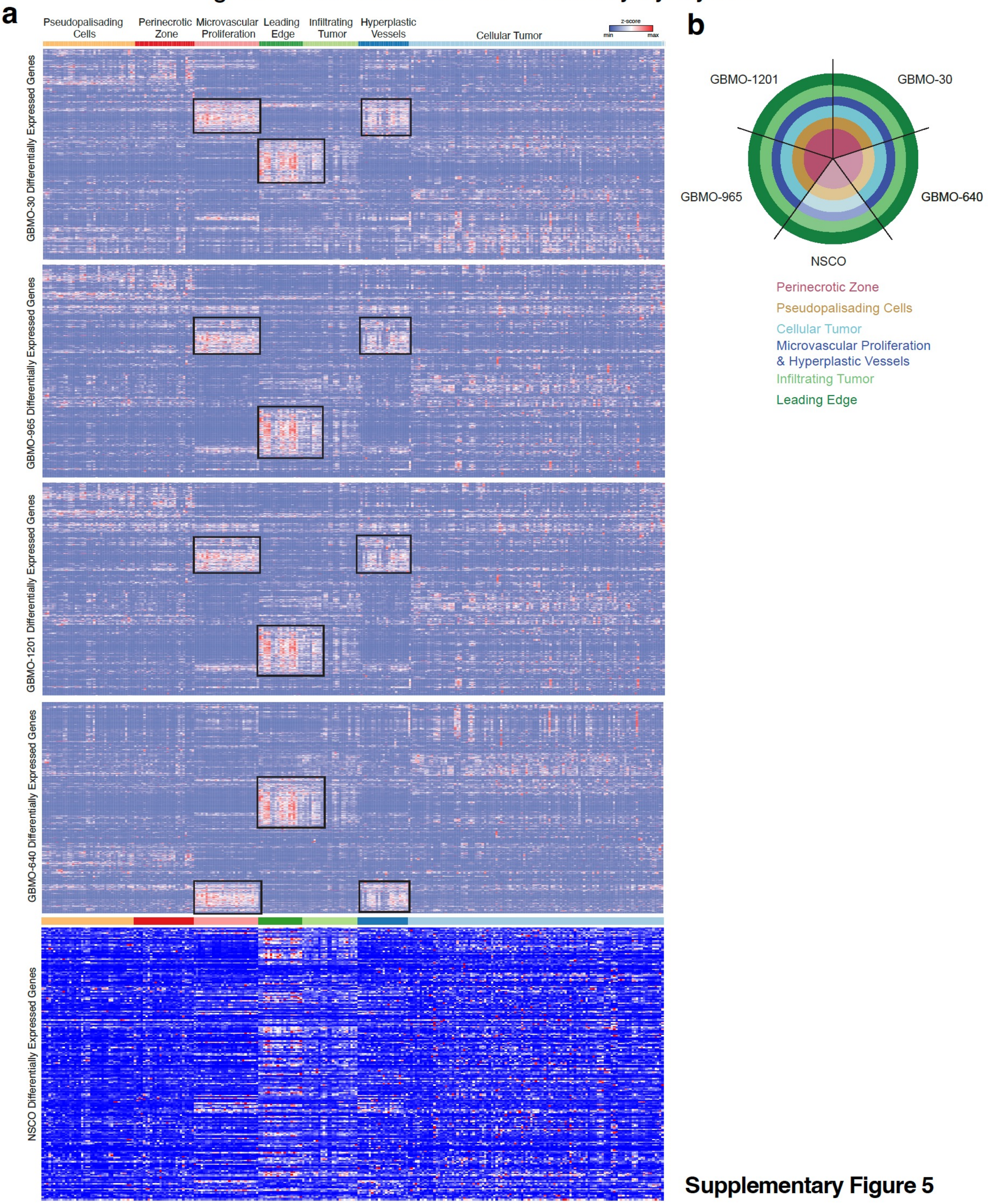

Supplementary Figure 5

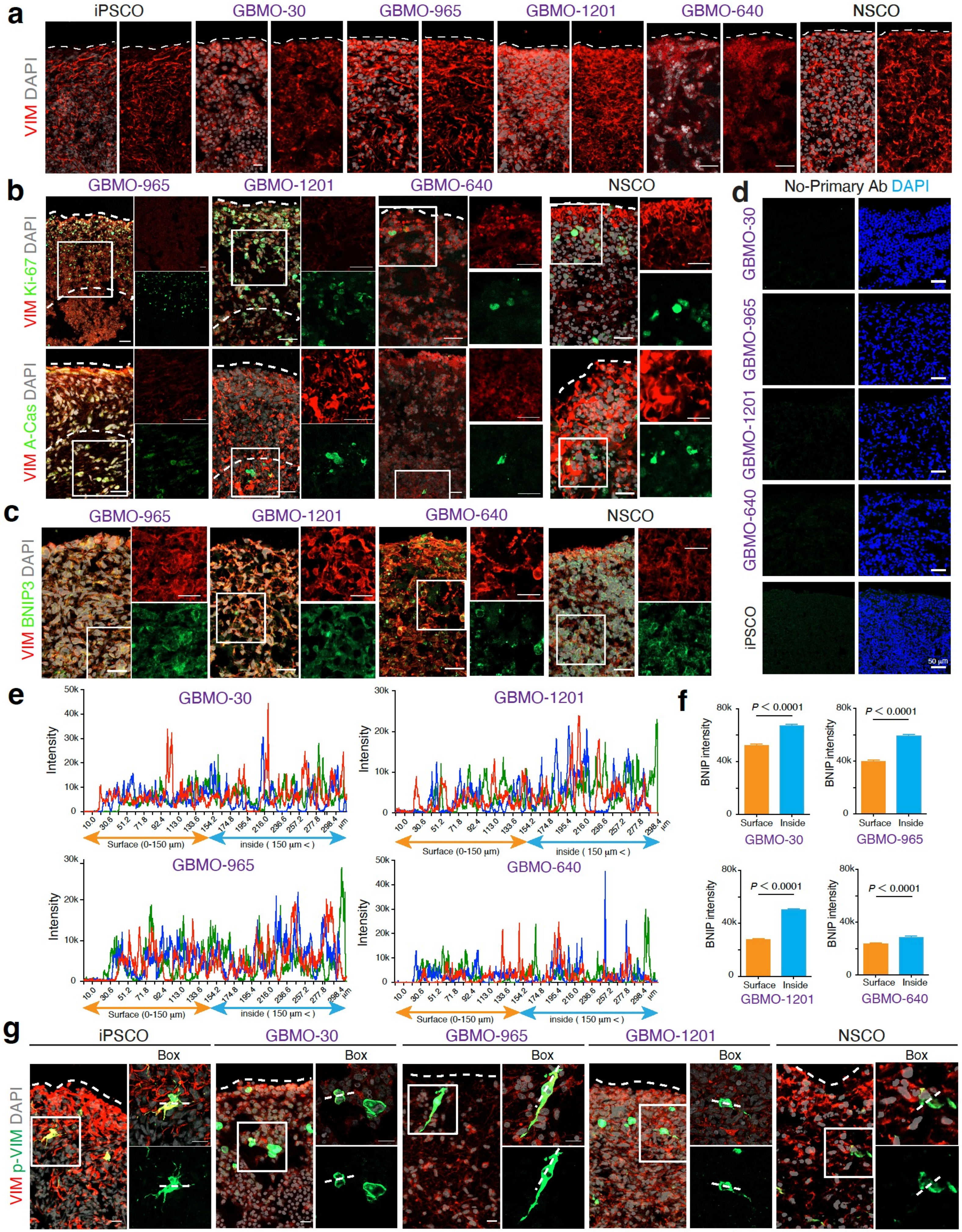

Supplementary Figure 6

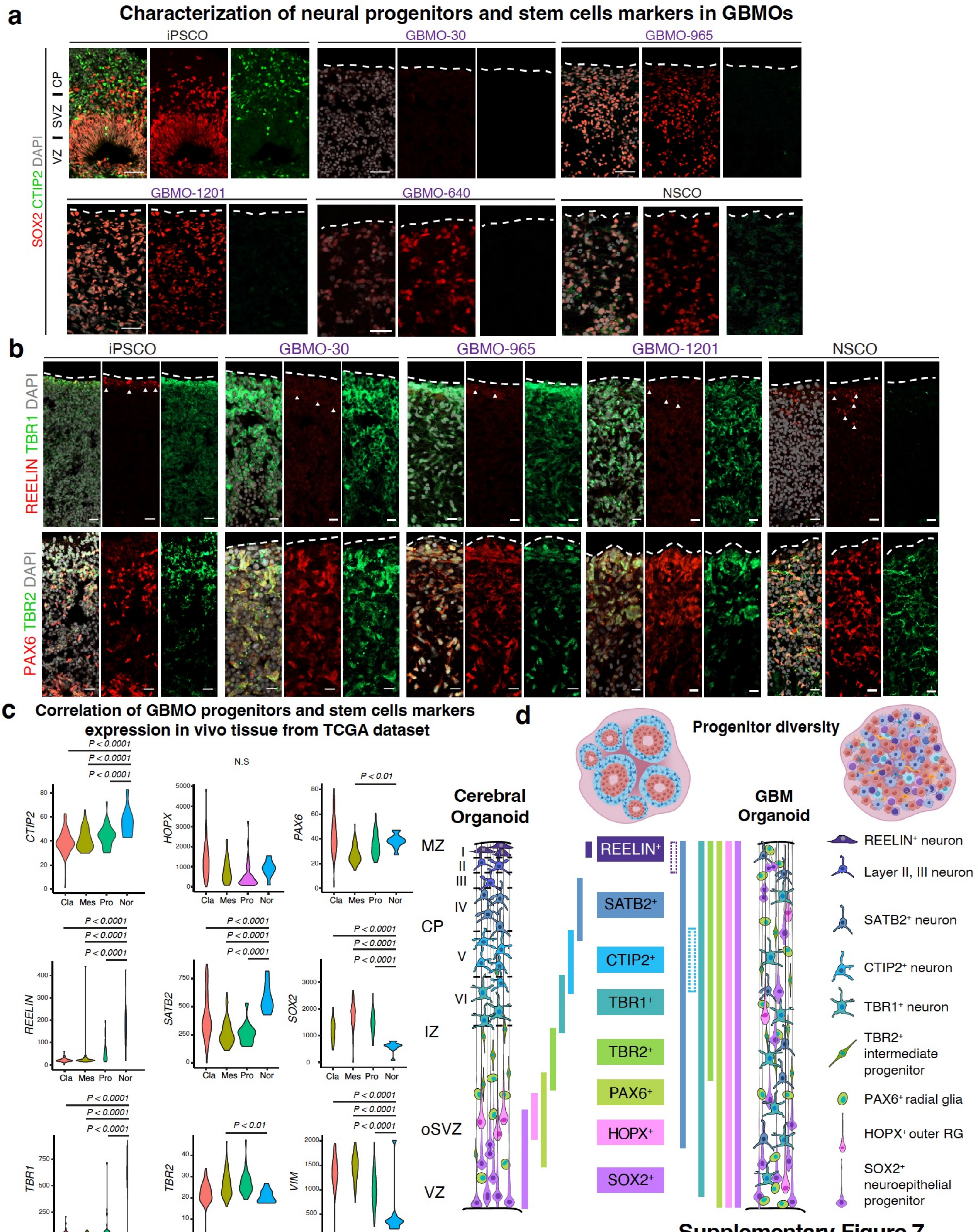

Supplementary Figure 7

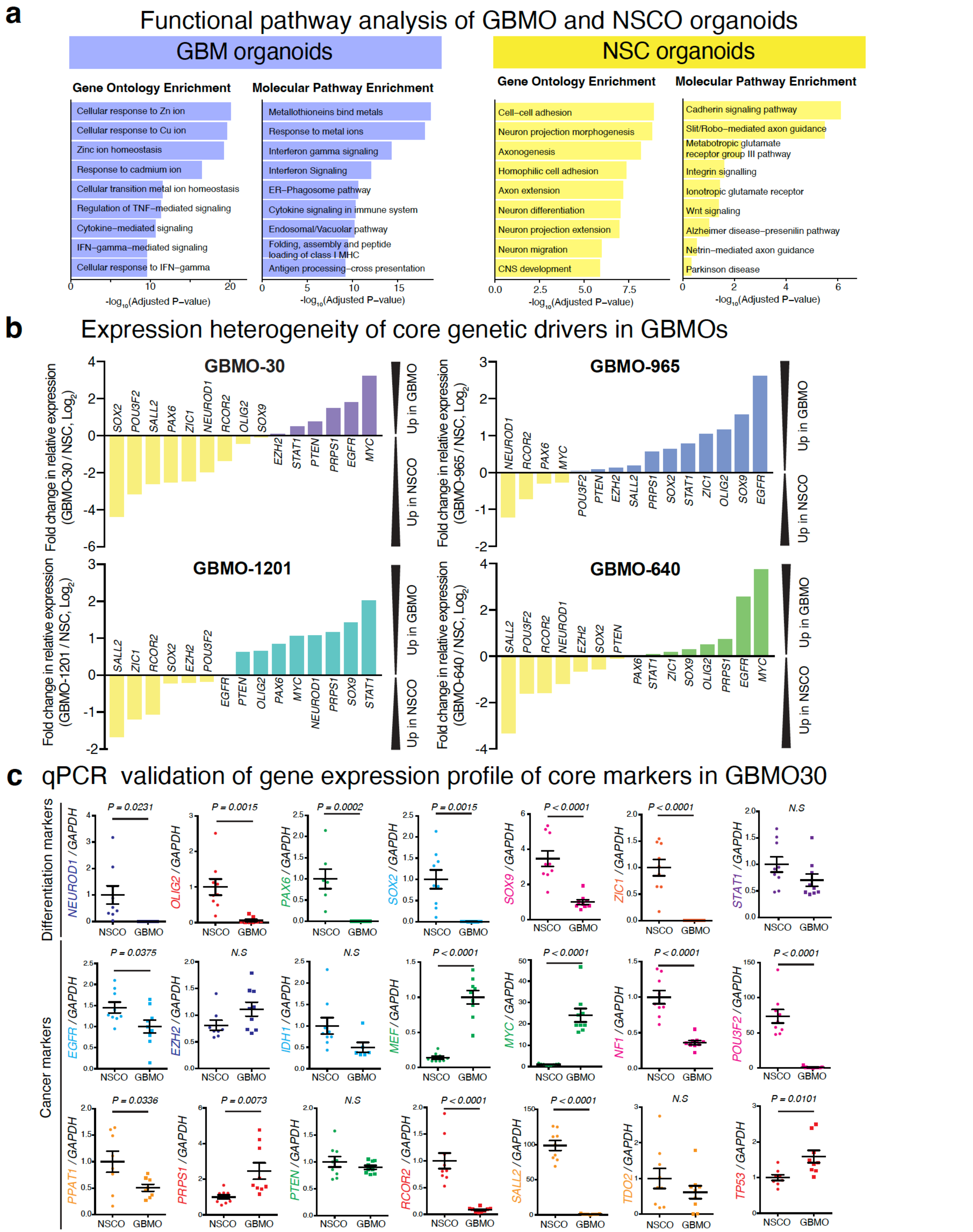

Supplementary Figure 8

a Validation of absence of absence of immunological cells in GBMOs

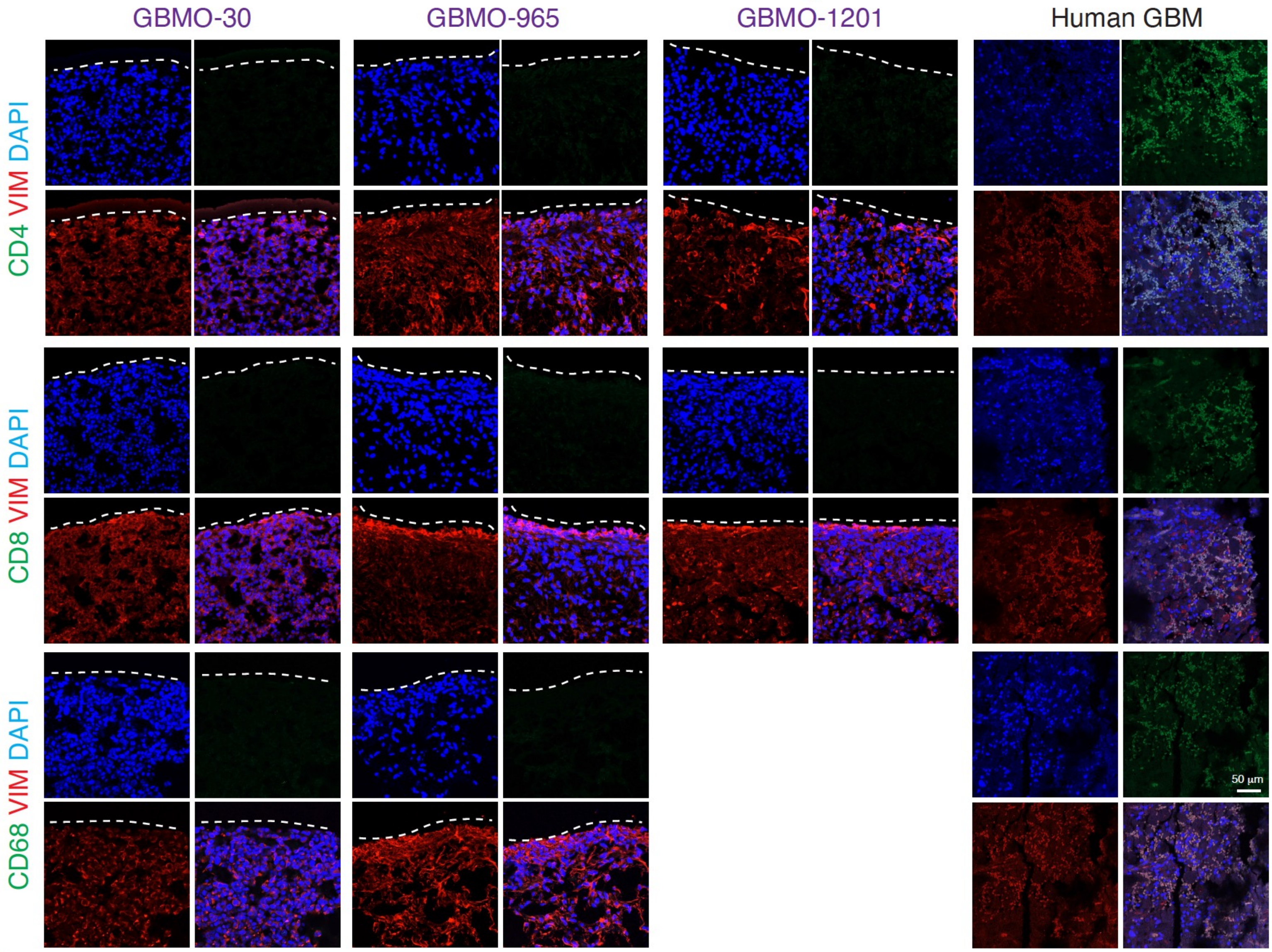

b Correlation of glioma intrinsic immune gene expression by single cell dataset

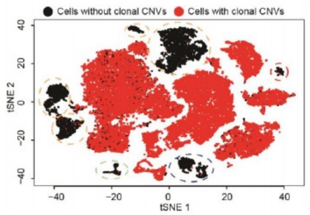

1. Glial-immune preferential expressed in tumor
2. Expressed in glial-immune and bona fide immune cells
3. Expressed only in bona fide immune cells

c Localization of GBMO immune associated gene expression in single cell dataset

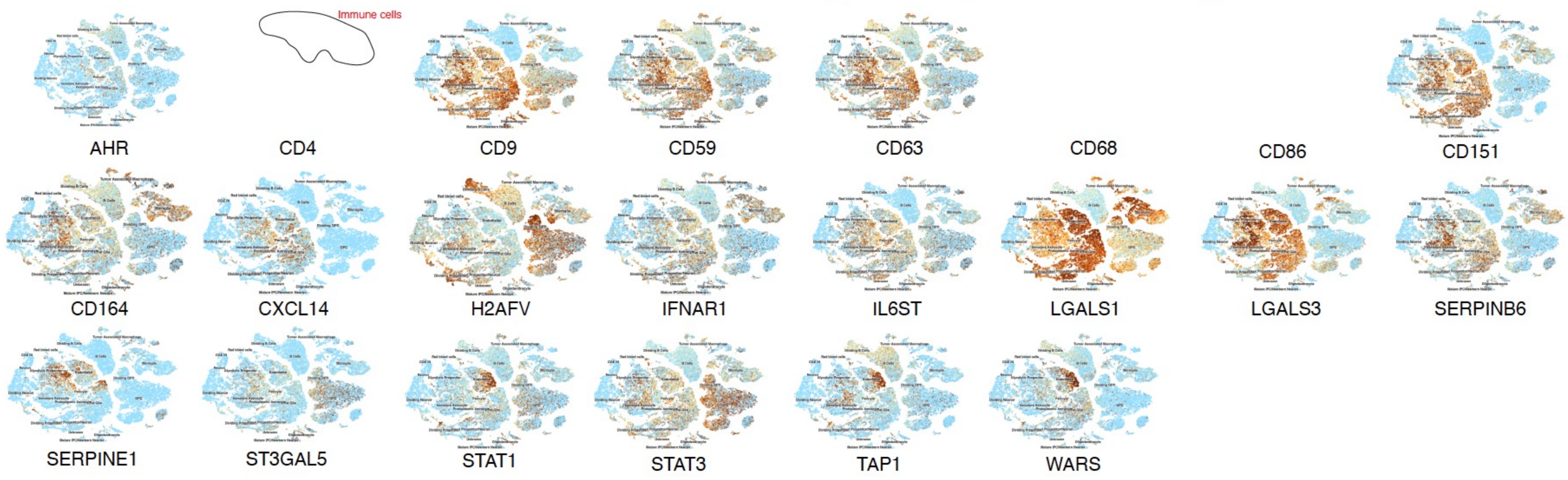

Supplementary Figure 9

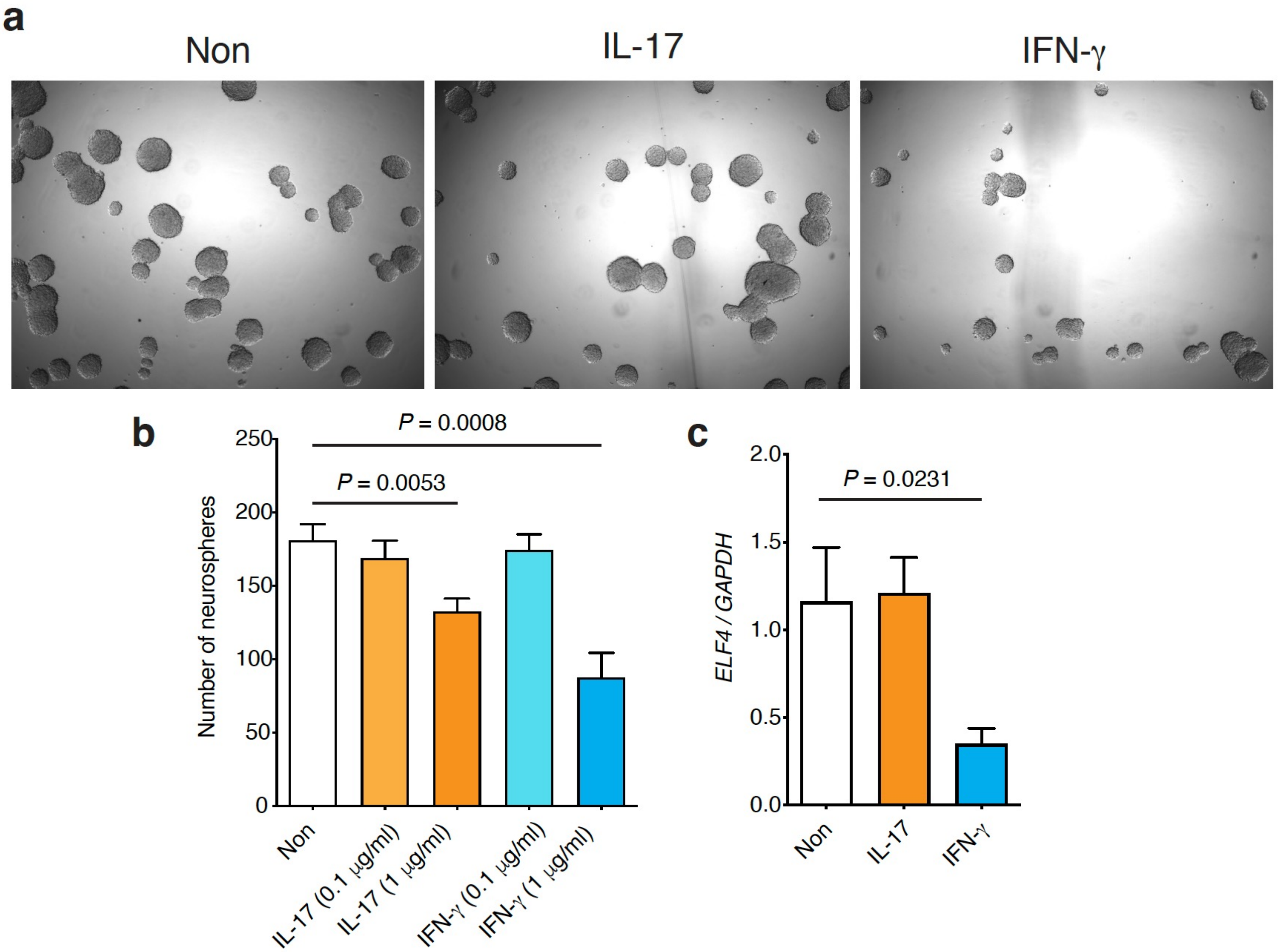

Supplementary Figure 10

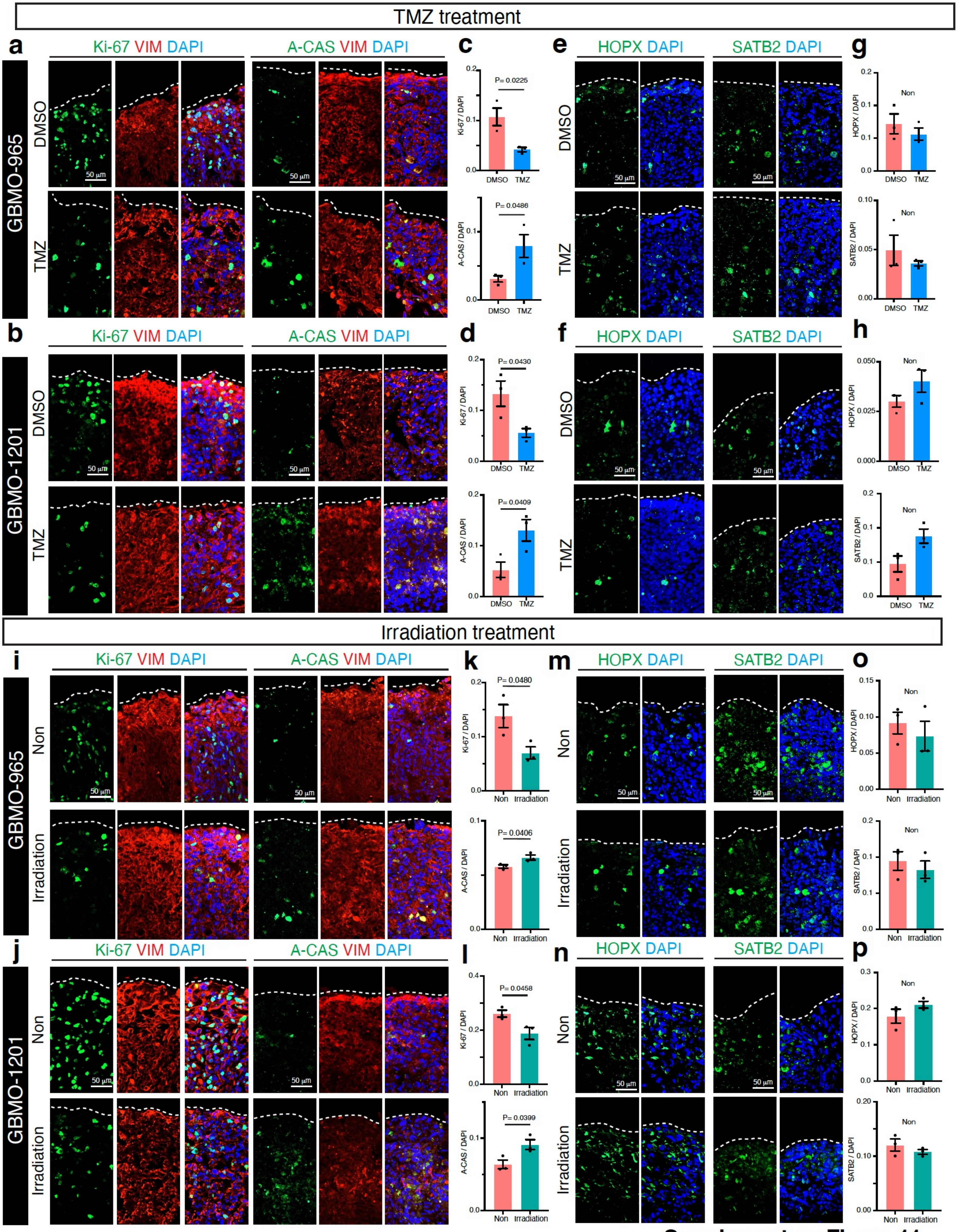

Supplementary Figure 11

Supplemental Data, Methods, Figures and Tables

Patient-Derived Organoids Recapitulate Glioma-Intrinsic Immune Program and Progenitor Populations of Glioblastoma.

Authors:
Fumihiro Watanabe[1,2#], Ethan W. Hollingsworth[1,2,10#], Jenna M. Bartley[3], Lauren Wisehart[1], Rahil Desai[1], Annalisa M. Hartlaub[4], Mark E. Hester[4,5,6], Paula Schiapparelli[7], Alfredo Quiñones-Hinojosa[7], and Jaime Imitola[1,2*]

Supplemental data

Detailed validation of the GBMO modelling progenitor diversity in GBM in the absence of infiltrating immune cells supplemental figures 1-

Patient-derived GSCs self-organize into 3D tumor tissue-like structures.

We cultured GSC-derived tumorspheres from patients who underwent GBM resection, all GSCs used in the study were authenticated and were free of *Mycoplasm sp* as shown in Methods **Supplementary Figure 1)**. We then generated GBMOs, that are proven to be GSCs *in vivo* and *in vitro* in prior work.[20-23] **Supplementary Figure 2a-c, Supplementary Table 1**). A prior report always maintained GSC-derived organoids under conditions of EGF/FGF-2. Typically, organoids are maintained in defined medium without added growth factors to form organoids, so we favored this method to allow comparison with control iPSCs organoids. We embedded GSCs in Matrigel and generated GBMOs, GSCs aggregated and integrated into a uniform organized tissue architecture. Pathology comparison of GBMOs and parental tumors showed similar proliferative and quiescent/necrotic compartments, in addition to the presence of pseudopalisading cells in selected tumor-organoid pairs (**Supplementary Fig. 2d,e**), For instance, GBMO-965 remained sparsely cellular with syncytia-like formations while GBMO-1201 retained its multicellularity with fibrils and occasional highly chromatic nuclei. Our GBMO generation had a 94% success rate (n=444 organoids from 6 GSC lines after 4 weeks in culture) (**Supplementary Fig. 2f**). The organoids reached sizes 20-fold larger than GSCs grown as “tumorspheres” (cancer cell aggregates), exhibit organization. In contrast, GSCs cultured as tumorspheres in EGF/FGF lacked any such organization (**Supplementary Fig. 3a,b**). These findings are similar to different protocol of GBM organoids procurement from intact tissue (GBOs) that reproduce features of their parental tumors[11]. These findings demonstrated that GSCs can form 3D organoids without added growth factors allowing us to perform in depth-phenotypic comparisons using similar cultures protocols for iPSCs (iPSCO) and organoids derived from NSCs (NSCO).

Genomic architecture of GBMO recapitulate alterations found in GBMs.

Thus far, detailed genomic and mutational analyses of GSC-derived GBMO have not been performed. To evaluate the genetic landscape of GBMO, we performed targeted massively parallel DNA sequencing (TMPDS) and large-scale chromosomal copy-number variation (CNV) analysis of all patient-derived GBMOs[48] and NSCO[16] that were cultured simultaneously with identical culture conditions **(Supplementary Fig. 4a).** CNV analysis revealed a greater number of CNVs across all GBMOs, relative to NSCO. These included sizable amplifications in

chromosome 7 and deletions in chromosome 10, which were shared among GBMO-1201, GBMO-965, and GBMO-640 and have been frequently reported in GBM (**Supplementary Fig. 4b and Supplementary Table 4**). We next employed TMPDS to identify somatic mutations in GBMO and NSCO. From a panel of 407 cancer-related genes, we identified 32 somatic mutations in GBMO-30, nine in GBMO-965, seven in GBMO-1201, and five in GBMO-640 while NSCO harbored five non-damaging mutations (**Supplementary Fig 4c and Supplementary Table 5**). Of the genes mutated in GBMO, the majority (GBMO-30, 70%; GBMO-965, 89%; GBMO-1201, 71%; GBMO-640, 60%) are mutated in native GBM *in vivo*[49]. Moreover, whereas four of the five mutated genes in NSCO are tolerated missense (SIFT score <0.05) (**Supplementary Fig 4d**), most GBMO mutations are predicted to be damaging, frame-shifting, or protein-truncating (**Fig. Supplementary Fig 4c-e**). GBMO-30, in particular, was composed of the most damaging frame-shift and nonsense mutations (**Supplementary Fig d,f**). This mutational spectrum, consisting of nonsynonymous mutations with an increased frequency of G>A/C>T transversions, is also in accord with that of GBM[50]. Functionally, many of the GBMO-detected mutations affect genes commonly mutated in GBM, including *IDH2*, *TP53*, and *PTEN*, the chromatin modifiers, *CREBBP*, *SETD2*, *KDM6A*[51], and *ATRX*, with the latter involved in impaired non-homologous end joining and found in 30% of GBM[52]. Altogether, these data demonstrate that GBMO mimic the high degree of aneuploidy, copy number changes, and somatic mutations[53] seen in GBM (**Supplementary Fig 4e,f and Supplementary Table 5**).

Molecular landscapes of GBMO recapitulate metabolic and molecular alterations found in GBM.

Next, we performed gene expression analysis in GBMO and NSCO in which both organoids were maintained in identical culture conditions. Principal component analysis of GBMO global transcriptomes indicated similarity between GBMO-1201 and GBMO-965 as well as between NSCO and GBMO-640, while GBMO-30 clustered by itself, suggesting a distinct expression state from the others **(Supplementary Fig 4g**). Using the classifier gene sets[54], we then calculated GBM subtype scores and found that GBMO-965, -640, and -1201 are most aligned with the classical subtype (**Supplementary Fig 4h**). GBMO-30 expressed predominantly mesenchymal subtype genes, in agreement with its absence of SOX2. Consistent with the high metabolic profile of GBM, differential expression analysis indicated that all GBMO except GBO-640 are driven by the Warburg Effect where aerobic glycolysis and lactate fermentation predominate (**Supplementary Fig 4i**). Additionally, we found that GBMO showed elevated methionine cycle gene expression, a recently uncovered metabolic pathway essential for glioma stem cells[55](**Supplementary Fig 4j**). Seahorse metabolic analysis confirmed elevated levels of mitochondrial ATP (Mito ATP) and glycolytic ATP (Glyco ATP) production rates in GBMO compared to NSCO (**Supplementary Fig 4k-m).**

Since we found distinct niches for proliferation, hypoxia, and quiescent cells in GBMO, we next asked how genes upregulated in GBMOs reflect different GBM histological compartments. To do this, we used the IVY Glioblastoma Atlas Project (Ivy GAP), an RNA sequencing dataset of microdissected GBM structures from 37 patients. While NSCO genes localized to the leading edge, which is known to contain endogenous neural and neoplastic tissue, we observed genes upregulated in GBMOs to be highly expressed in the leading edge, hyperplastic vessel, and microvascular proliferative domains that are populated by malignant migrating cells (**Supplementary Fig.6 and Supplementary Table 10, 11, 12, and 13**).

Supplemental Methods

Generation of organoids and cell culture.

Human GBM stem cells (GSCs) were procured from patients and allocated for human research purposes, per the protocols approved by the Institutional Review Boards (IRB) at The Ohio State University Wexner Medical Center and Mayo Clinic[24,25]. Confirmatory properties as GSCs for these lines has been demonstrated elsewhere (**Supplementary Table 1**). All GSCs used in the study were authenticated and were free of *Mycoplasm sp* (**Supplementary Figure 1)**. Patient-derived GSCs were isolated and cultured in Neurobasal, 2% B27 supplement without Vitamin A, Glutamax, Antibiotic-Antimycotic (Thermo Fisher Scientific, Waltham, MA, USA), 50 ng/ml human epidermal growth factor (EGF), 50 ng/ml basic fibroblast growth factor (FGF) (R&D Systems, Minneapolis, MN, USA), and heparin (Millipore Sigma, Billerica, MA, USA) in low-attachment cell culture flasks, as previously described[14]. Human neural stem cells (NSC, H9 hESC-Derived, Invitrogen) were cultured on Geltrex in KnockOut DMEM/F12, 2% StemPro Neural supplement, Glutamax, Antibiotic-Antimycotic (Thermo Fisher Scientific), 50 ng/ml human EGF, 50 ng/ml human FGF in low-attachment cell culture flasks, several subclones were generated for the study. For GBM and NSC organoid formation, dissociated single cells were cultured with GBM or NSC growth media for 4-7 days. GBM or normal neurospheres were transferred to Matrigel droplets (BD Bioscience, San Jose, CA, USA) by pipetting into cold Matrigel on a sheet of Parafilm with 3 mm dimples. These droplets were allowed to gel at 37°C and were subsequently removed from the Parafilm. After 4 days of stationary growth, the tissue droplets were transferred to a spinning bioreactor containing organoid differentiation media including 48% Neurobasal, 48% DMEM/F12, 1% B27 supplement, 0.5% N2 supplement, 1% Glutamax, 0.5% MEM-NEAA, and 1% Antibiotic-Antimycotic[55]. Human iPSCs and iPSC-derived organoids were generated as described previously[55]. All cell lines were handled in accordance with the IBC biosafety practices and relevant ethical guidelines of The Ohio State University College of Medicine, Nationwide Children's Hospital, and University of Connecticut that regulate the use of human cells for research.

Histology and immunofluorescence.

Tissues were fixed in 4% paraformaldehyde for 20 min at 4˚C followed by washing in PBS three times for 10 min. Tissues were allowed to sink in 30% sucrose overnight, embedded in OCT compound (Tissue-Tek, Sakura Finetek USA, Torrance, CA, USA), and then cryosectioned at 20 μm. Tissue sections were stained with hematoxylin and eosin, and images were taken with a light microscope (BX41, Olympus, Tokyo, Japan) equipped with a digital camera (DP71, Olympus). For immunofluorescence, sections were incubated successively with 0.25% Triton X-100 and 4% normal horse serum in PBS for 30 min, primary antibodies overnight, and Alexa Fluor 488-, 594-, or 647-conjugated species-specific secondary antibodies for 2 h (Thermo Fisher Scientific). Vectashield Mounting Medium with DAPI (Vector Laboratories, Burlingame, CA, USA) or DAPI (Thermo Fisher Scientific) were used for counterstaining. For single labeling of tissue, the following primary antibodies against the following molecules (immunized species) were used: activated-caspase-3 (559565, BD Pharmingen, San Jose, CA, USA), AHR (ab190797 and ab2769, Abcam), BNIP3 (sc-56167, Santa Cruz Biotechnology), CD4 (550278, BD Pharmingen), CD68 (556059, BD Pharmingen), CD8 (sc-18913, Santa Cruz), CTIP2 (ab18465, Abcam), HOPX (HPA030180, Millipore Sigma), IFNGR1 (MABF753, MilliporeSigma), Ki-67 (ab16667, Abcam), PAX6 (AB_528427, DSHB, Iowa city, IA, USA), phosphorylated-vimentin (D076-3, MBL, Nagoya, Japan), REELIN (MAB5366, Millipore Sigma), SATB2 (ab92446, Abcam), SOX2 (AF2018, R&D systems), STAT1 (610185, BD Bioscience), TBR1 (ab31940, Abcam), TBR2 (ab23345, Abcam), and vimentin (sc7557, Santa Cruz). Images were taken with a confocal laser-scanning microscope (LSM800, Carl Zeiss Microscopy GmbH, Jena, Germany).

Quantitative analysis of immunostaining.

Markers for proliferation and apoptosis (Ki-67 and activated-caspase-3) were quantified and normalized with respect to nuclear DAPI staining, on the outer and inner surface areas of organoid images. Using ImageJ software, quantification of marker staining was performed in 3 equally sized rectangular areas that were overlayed on each outer and inner surface. A minimum of three sections were quantified for each organoid line. The length of relative layer thickness was measured using ImageJ software.

Targeted parallel sequencing and CNV analysis.

A custom capture-based, targeted next-generation sequencing panel, which includes probes covering the coding sequences of 407 cancer-related genes and genome-wide copy number variation (CNV) of backbone targets (Agilent OneSeq 300kb CNV Backbone + custom panel), was utilized in this study. Sequencing libraries were produced using standard methods, barcoded, and sequenced in pools on a HiSeq4000 by the OSU-CCC Solid Tumor Translational Science Shared Resource. Experimental DNA samples were run side-by-side with a human reference DNA sample. Raw sequence reads were aligned with bwa-0.7.13[56] aln and sample to Homo sapiens genome 1000g v37. Alignment was converted to bam with samtools v1.3.1[57]. After adding read groups, marking duplicates, and sorting with picard 2.4.1[58], Genome Analysis Toolkit (GATK) v3.6[59] was used to realign around indels. Mpileup format was generated with samtools v1.3.1 requiring quality > 1 and varscan v2.4.1[60] ProcessSomatic and Variant Effect Predictor from ensembl[61] were used to identify tumor-specific variants for each sample. Variants were filtered for location (excluding non-coding variants), coding impact (excluding LOW impact variants – those unlikely to change protein behavior), allele frequency in public databases (excluding common variants found in 1000g[62] and ExAC[63]), and known variants (excluding variants listed in NCBI dbSNP[64] but not in COSMIC databases[65]). CNVkit, a python-based copy number calling software[66], was used to detect the copy number alterations from the samples using a single human "normal" sample as the reference. The log r ratios were calculated for both target and anti-target bins across the genome. Segmentation was performed on the binned data using the circular binary segmentation algorithm. For the heat maps, segments with medians greater than 0.25 were considered duplications and those with medians less than -0.5 were considered deletions. **For coverage metrics please Supplementary tables 2, 3.**

Real-Time Quantitative PCR.

RNAs were extracted from cell cultures with QIAzol reagent and miRNeasy Mini Kit (QIAGEN, GmbH, Hilden, Germany), following the manufacturer’s protocol. cDNAs were obtained from 500 ng of mRNA using the retrotranscription kit (Thermo Fisher Scientific). Quantitative real-time PCR was performed on 1/20 of the retrotranscription reaction using SYBR Green PCR Master Mix (Thermo Fisher Scientific). Primers were designed to amplify 50- to 200-bp fragments; All primer sequences can be found in **Supplementary Table 14**. The qPCR data were assessed using delta-delta CT for evaluating results in the sigmoid region of the amplification curve. For each analysis, samples were normalized by comparison with the housekeeping gene GAPDH. All samples, including “no template” controls, were assayed in triplicate. Each experiment was performed three times with comparable results. Data are expressed as mean ± SEM.

Transcriptome analysis and differential expression.

The GeneChip Human Transcriptome Array 1.0 (also known as Clariom D assays; Affymetrix, Thermo Fisher Scientific Inc.) was used to provide a detailed analysis of the organoid transcriptome. Briefly, 100 ng of total RNA from each of the three samples originally assigned for microarray analysis were used to generate amplified and biotinylated sense-strand cDNA from the entire expressed genome according to the GeneChip WT PLUS Reagent Kit User Manual

(P/N 703174, Affymetrix Inc., Santa Clara, CA). cDNA was hybridized to GeneChip Human Transcriptome Array 1.0 for 16 hr in a 45°C incubator, rotated at 60 rpm. After hybridization, the microarrays were washed, and then stained using the Fluidics Station 450 followed by scanning with the Affymetrix GeneChip Scanner 3000 7G, according to manufacturer's instructions. Raw intensity data was normalized using the quintile normalization of robust multiarray average (RMA) method (performed at the individual probe level). Probes with low variance were filtered out using the R package genefilter[67] and annotated to the human genome using the Human Clariom D platform. Transcripts were identified as differentially expressed using the limma package[68], with a threshold of FDR-adjusted p-value <0.05 and fold change greater than ±2.

Principal component and subtype analysis, hierarchal clustering, and functional annotation.

We reduced the dimensionality of the data by performing principal component analysis (PCA) on the organoid microarray datasets using the prcomp function in R (center = TRUE, scale = FALSE), including only the filtered genes with moderate to high variance. The average relative expression of each set of GBM subtype predictor genes (as defined by TCGA[36] was quantified from the log2-transformed expression values to determine a relative subtype score for defining each GBMO line. Subtype average expression was normalized by the average log2 expression value of all subtype genes. To identify potential transcriptional modules based on the co-expression of genes in the organoid dataset, unsupervised hierarchical clustering of differentially expressed genes (DEGs) was performed using average linkage and uncentered Pearson correlation on variably expressed genes, as determined by the varFilter() function in limma. Log2-scaled expression values were centered on the median before performing hierarchical clustering. Heatmaps of clustered differential gene expression were then generated. Differentially expressed gene data and the resulting clusters (FC±2, p<0.05) were exported for further functional analysis. We made use of Enrichr, which utilizes the Fisher exact test with multiple hypothesis testing correction[69], to determine gene ontologies, dysregulated pathways, and predicted upstream transcription factors.

Unbiased search strategy for GSC molecular vulnerabilities.

To establish an unbiased GBMO-intrinsic genetic program, normalized intensity values for each GBMO line were first filtered to include only the top one-third of highly expressed genes. The resulting gene lists were compared among GBMO lines and the genes shared by all four lines were the only ones further considered. Gene lists were inputted into Enrichr for enrichment analyses[46]. To further ascertain the immune expression states of each GBMO line, we concentrated on the expression of immune-associated genes, as obtained from ImmPort (http://www.immport.org/immport-open/public/home/home) and InnateDB (http://www.innatedb.com)[70], using the original DE analyses to ensure all IAGs were encompassed. Plots were produced using ggplot2 package in R. Again, the convergence of the DE IAGs was utilized and then filtered to focus only on known human transcription factors, as specified by http://fiserlab.org/tf2dna_db//index.html. For heatmap generation, data were imported into the online matrix software, Morpheus (https://software.broadinstitute.org/morpheus).

Processing of single-cell RNA-seq datasets from GBM *in vivo*.

Raw read count matrices for Neftel et al[71] and Muller et al (https://www.biorxiv.org/content/10.1101/377606v1.full[72]) were downloaded from GSE131928 and UCSC Single Cell Browser (https://cells.ucsc.edu/), respectively. A Seurat object was created for each matrix separately and datasets were scaled. The expression of *HOPX* and *SATB2* were then visualized. In each dataset, an expression cut-off of 50% of maximal gene expression was

established and all cells expressing at or above this were deemed to be $HOPX^+$ or $SATB2^+$. Since we found expression of these genes were not mutually exclusive, we created a third category, $HOPX^+SATB2^+$, for cells expressing both these genes. Cell populations were identified according to these population criteria and the expression of well-known immune associated genes visualized. Upregulated genes in each cell population were then determined for each population using the FindAllMarkers command of Seurat v3 [73]. From these upregulated gene lists, pathway analysis was performed using Enrichr. After noting an enrichment of immune-related genes across both datasets, we extracted interferon stimulated genes from the upregulated interferon stimulated genes. We then made use of StringDB (https://string-db.org/) to generate protein-protein interaction networks for the top-25 upregulated ISGs of each cell population and merged these networks from each dataset together.

Retrospective analysis of gene expression in human gliomas.

Gene expression of neurodevelopmental progenitor markers and upregulated GBMO genes were determined across primary patient gliomas and subtypes of GBM tumors, determined through analysis of the National Cancer Institute Repository for Molecular Brain Neoplasia Data (http://betastasis.com/glioma/rembrandt/) and TCGA (https://tcga-data.nci.nih.gov/publications/tcga), respectively. Gene expression localization in structures of primary patient GBM was determined through analysis of Allen Institute of Ivy GAP (http://glioblastoma.alleninstitute.org/). Expression data was downloaded and heatmaps were generated using the matrix visualization software, Morpheus. Heatmaps of Pearson similarity matrices were also generated using Morpheus. Detailed information of heatmaps, including gene names in retained order as in **Supplementary Fig. 5** can be found in **Supplementary Tables 10-14**.

Metabolic analysis of GBMO with Seahorse technology.

For Seahorse Analysis (XFe96, Agilent Technologies), organoids were first dissociated via dissociation reagent. Dissociated cells were washed into warmed Seahorse XF DMEM medium supplemented with 10 mM glucose, 1 mM pyruvate, and 2 mM glutamine and plated at a density of $1x10^5$ cells/well on a poly-L-lysine coated XFe96 Seahorse cell culture microplate. Cells were simultaneously tested for oxygen consumption rate (OCR) and extracellular acidification rate (ECAR) per the manufacturer's XF Real-Time ATP Rate Assay Kit protocol. Mitochondrial ATP (mitoATP) and glycolytic ATP (glycoATP) production rates were calculated via Agilent Seahorse XF Real-Time ATP Rate Assay Report Generator. ATP Production Rates were analyzed via t-test or ANOVA with Bonferroni posthoc corrections as needed, with significance set at $p<0.05$.

Organoid generation with Chemotherapeutic agents or irradiation.

To determine the effect of chemotherapeutic agents for GSC-derived organoid, organoids were cultured as previously shown. After 4 days of stationary growth, the tissue droplets were transferred to a spinning bioreactor containing organoid differentiation media including 48% Neurobasal, 48% DMEM/F12, 1% B27 supplement, 0.5% N2 supplement, 1% Glutamax, 0.5% MEM-NEAA, and 1% Antibiotic-Antimycotic then cultured in the presence of the Temozolomide (TMZ, 30 and 300 μM). To determine the effect of irradiation for GSC-derived organoid, small neurosphere of GSCs were treated with irradiation (10 Gy). The irradiation of cells was performed with the gammecell irradiator. And then organoids were cultured as previously shown.

SUPPLEMENTARY FIGURE LEGENDS

Supplementary Figure 1. Authentication of cell lines
Report of authentication of cell lines, all GSCs used in the study were authenticated and were free of *Mycoplasm sp*.

Supplementary Figure 2. Selected Patient MRI and histopathology of parental tumors and corresponding GBMO. (a, b) Coronal and sagittal MRI slices and tissue pathology of brain tumor patients #965 (left) and #1201 (right). **(c)** Experimental procedure from resected brain tumor to GBM organoids via glioblastoma stem cells (GSCs). **(d, e)** Histological analysis by hematoxylin and eosin (H&E) staining using parental tumor tissue and patient-derived GBM organoid 965 (GBMO-965, left) and 1201(GBMO-1201, right). Red arrows, elongated cells; Orange arrows, syncytia; Blue arrows, fibrils; Green arrows, fusiform cell clusters; Yellow arrows, cells with clear cytoplasm; Black arrows, highly chromatic nuclei. Scale bars, 100 µm. **(f)** Success rate for generating GBMO in different patient derived-glioblastoma stem cells, a total of 444 organoids were generated.

Supplementary Figure 3. Comparison of in vitro modeling of GBM biology between GBMO and GSC tumorspheres. (a) Comparison of features between GBM tumorsphere and GBM organoid (GBMO). **(b)** Immunocytochemistry for Ki-67, active-caspase-3 (A-Cas), HIF-1α, and vimentin with GBM-30 GSCs on poly-lysine (2D culture). Bar graphs are presented as mean ± SEM. Two-tailed unpaired Student's *t*-test (N.S. = not significant, $P > 0.05$).

Supplementary Figure 4. Mutational, transcriptomic, and functional metabolic landscape of patient derived GBMO. (a) Diagram of genomic sequencing and microarray analysis for GSC-derived glioblastoma organoid intrinsic molecular phenotypes. **(b)** Large-scale copy number variant (CNV) analysis of GBMO-1201, -640, -965, -30, and NSCO (-01, -02 are technical replicates from different batches). Red, amplifications; blue, deletions. **(c)** Mutational classification in GBMO lines and NSCO, as defined by Catalogue of Somatic Mutations in Cancer (COSMIC). **(d)** Summary of somatic mutation types and functional classification of mutated genes in each GBMO line and NSCO. Complete details are found in **Supplemental Table 3**. **(e)** Quantification of mutation types indicates an enrichment of damaging, coding mutations in GBMO-30, -965, -1201, -640, relative to NSCO. Indel, insertion and/or deletion. **(f)** Transition/transversion (Ti/Tv) proportion comprising single-nucleotide variants for GBMO-30, -965, -1201, -640, and NSCOs, as identified by TPDS. **(g)** Principal component analysis (PCA) of GBMO and NSCO transcriptomes. **(h)** Relative expression of classifier genes belonging to each GBM molecular subtype. **(i)** Metabolomic profiles, as inferred from transcriptomic analysis, of each GBMO line and adjoining summary schematic of classical biochemical pathways. 1,3-BPG, 1,3 Bisphosphoglycerate; F-1,6-BP, Fructose-1-6-Bisphosphate G-6-P, Glucose-6-Phosphate; PPP, Pentose Phosphate Pathway; TCA, Tricarboxylic Acid. **(j)** Expression and schematic of genes involved in the methionine cycle in GBMO and NSCO. GBMO-30, GBMO-965, and GBMO-1201). **(k)** Metabolic analysis with Seahorse XF Real-Time ATP Rate Assay of GBMO-30, 965, 1201 and hNSCO showing mitochondrial ATP (Mito ATP) and glycolytic ATP (Glyco ATP) production rates. Total ATP production: $p<0.0001$, mito ATP: $p<0.0001$, glycol ATP: $p<0.0001$. **(l)** Oxygen Consumption Rate (OCR) and **(m)** Extracellular Acidification Rate (ECAR) were measured among hNSCO and each GBMO.

Supplementary Figure 5. Spatially resolved heatmap of GBMO-upregulated gene expression in GBM microstructures in vivo. (a) Heatmap depicting the spatial expression of upregulated genes in GBMO-30, -965, -1201, and -640 by tumor microanatomy. Complete details of gene names can be found in Supplementary Table 3. RNA-Seq data of tumor structures is

derived from IvyGAP. **(b)** Summary figure for these heatmaps. The colors were matched in the circle with the structure colors from the heatmap. Dark colors mean high expression; light colors mean less expression.

Supplementary Figure 6. Additional characterization of proliferation, apoptosis, and hypoxia in additional GBMO and hNSCO. (**a**) Representative images of vimentin (VIM) immunostaining in patient-derived organoid from GBM 30, 965, 1201 and 640 (GBMO-30, GBMO-965, GBMO-1201, and GBMO-640) and an organoid derived from human neural stem cells (hNSCO) and human induced pluripotent stem cells (iPSCO). Scale bar, 20 μm. (**b**) Immunohistochemistry for proliferative (Ki-67) and apoptotic (A-Cas) markers in GBMO-965, GBM-1201, GBMO-640, and hNSCO. Scale bar, 50 μm. **(c)** Immunohistochemistry for hypoxia marker (BNIP3) in GBMO-965, GBM-1201, GBMO-640, and hNSCO. Scale bar, 50 μm. **(d)** Negative control (no primary antibody) for each GBMO and iPSCO. **(e)** Hypoxia level (BNIP3 intensity) from surface to inside in GBM-30, -965, -1201, and -640. **(f)** Quantitative analysis for BNIP3 marker intensity between surface (0-150 μm) and inside (>150 μm from surface). **(g)** Sample images of immunostaining for VIM and p-VIM : markers of outer radial glia in iPSCO, GBMO-30, -965, -1201, and neural stem cell organoid (NSCO). Dotted lines highlight cleavage furrow of dividing cells. Scale bar, 20 μm.

Supplementary Figure 7. Characterization of markers for progenitor diversity in GBMO and their expression in vivo. (a) Immunostaining for the stem cell marker SOX2 and deep-layer neuron marker CTIP2 in iPSCO, NSCO, and GBMO lines. Scale bar, 50 μm. **(b)** Representative confocal images for immunostaining of Cajal-Retzius cell (top: REELIN), deep-layer cortical neuron (top: TBR1), intermediate progenitor (second: TBR2), and radial glia (second: PAX6) markers in iPSCO, hNSO, and each GBMO line. Scale bar, 20 μm. **(c)** Violin plots of marker mRNA expression by molecular subtype using publicly available TCGA cohorts. Cla, Classical; Mes, Mesenchymal; Pro, Proneural; and Nor, Normal. One-way ANOVA followed by Dunnett's correction, relative to Normal. N.S. = not significant, $P > 0.05$. Significant $P < 0.05$. **(d)** Schematic of tissue-like organization and progenitor diversity in iPSCO and GBMO, color bands represent the localization or absence of specific progenitor organized in layers in the normal organoids compared to glioblastoma organoids.

Supplementary Figure 8. Transcriptomic enrichment for immunity and cancer-related genes in GBMO with confirmatory qPCR in GBMO and NSCO. (a) Pathway enrichment using differentially expressed genes from GBMO (left) and NSCO (right). **(b)** Waterfall plot showing expression of notable genes between GBMO-30, -965, -1201, and -640 and NSCO, as detected by transcriptomic analysis. **(c)** Confirmatory qPCR indicates relative expression of differentiation marker genes (top) and cancer-related genes (bottom) between GBMO and NSCO by qPCR. Data are presented as means ± SEM. Two-tailed unpaired Student's t-test. (*NEUROD1*, $P = 0.0231$; *OLIG2*, $P = 0.0015$; *PAX6*, $P = 0.0002$; *SOX2*, $P = 0.0015$; *SOX9*, $P < 0.0001$; *ZIC1*, $P < 0.0001$; *EGFR*, $P = 0.0375$; *MEF*, $P < 0.0001$; *MYC*, $P < 0.0001$; *NF1*, $P < 0.0001$; *POU3F2*, $P < 0.0001$; *PPAT1*, $P = 0.0336$; *PRPS1*, $P = 0.0073$; *RCOR2*, $P < 0.0001$; *SALL2*, $P < 0.0001$; *TP53*, $P = 0.0101$.). N.S. = not significant, $P > 0.05$.

Supplementary Figure 9. Gene expression for immune system molecules from GBM single-cell RNA-seq and immunostaining for immune cell markers GBMO-30, GBMO-965, and GBMO-1201 and resected GBM tissue. (a) Representative confocal images for immunostaining of CD4 (top), CD8 (middle), and CD68 (bottom) in each GBMO line and primary human GBM (positive control). Scale bar, 50 μm. **(b)** Expression of immune-like molecules in the glioma cell

vs. infiltrating T cells or macrophages. **(c)** Gene expression in a GBM single-cell RNA-seq for molecules of immune system in vivo GBM.

Supplementary Figure 10. Effects of cytokine in GBMO-forming GSCs self-renewal and *MEF/ELF4* gene expression. (a) Neurosphere formation of GBM-30 with IL-17 and IFN-γ. **(b)** Quantification of neurospheres number among IL-17 and IFN-γ -treated GBM-30 GSCs. **(c)** Gene expression of *MEF/ELF4* in GBM-30 with IL-17 and IFN-γ.

Supplementary Figure 11. Effects of Temozolamide or irradiation on GBMO-965 and -1201 growth. (a, b) Immunofluorescent images for proliferation(Ki-67, left) and apoptosis (A-CAS, right) in GBMO-965 (a) and -1201 (b) with DMSO control (upper) and TMZ (lower). **(c, d)** Quantitative analysis for Ki-67 positive cells (upper) and A-CAS positive cell (lower). in GBMO-965 (c) and -1201(d) with DMSO control and TMZ treatment. **(e,f)** Immunostaining for radial glia (HOPX, left) and neural differentiation (SATB2, right) in GBMO-965 (e) and -1201(f) with DMSO control (upper) and TMZ (lower). **(g,h)** Quantitative analysis for HOPX-positive cells (upper) and SATB2-positive cell (lower) in GBMO-965 (g) and -1201 (h) with DMSO control (upper) and TMZ (lower). **(I, j)** Immuno staining for Ki-67 (left) and A-CAS (right) in GBMO-965 (i) and -1201(j) with control (upper) and Irradiation (lower). **(k,l)** Quantitative analysis for Ki-67 positive cells (upper) and A-CAS positive cell (lower) in GBMO-965 (k) and-1201 (l) with control (upper) and irradiation (lower). **(m,n)** Immunofluorescence for HOPX (left) and SATB2 (right) in GBMO-965 (m) and-1201 (n) with control (upper) and Irradiation (lower). **(o,p)** Quantitative analysis for HOPX-positive cells (upper) and SATB2-positive cell (lower) in GBMO-965 (o) and-1201 (p) with control (upper) and Irradiation (lower).

SUPPLEMENTARY TABLE INDEXs.

SUPPLEMENTARY TABLE 1. Clinical Information Describing Patient Donors of Primary Glioblastoma Cells, Related to Figure 1.

SUPPLEMENTARY TABLE 2. Specific Coverage Metrics for Targeted Panel Sequencing and Copy-Number Variation, Related to Figure 2. HumRef, human reference; Coverage ratio = Targeted Panel:CNV

SUPPLEMENTARY TABLE 3. Genomic Coverage Metrics for Sequencing, Related to Figure 2.

SUPPLEMENTARY TABLE 4. Wide-Scale Copy Number Alterations of GBMO-30 and NSCO, Related to Figure 2.

SUPPLEMENTARY TABLE 5. Somatic Mutation Summary of GBMO-30 and NSCO, Related to Figure 3. LC, Low confidence; bolded gene names have publication(s) in glioma.

SUPPLEMENTARY TABLE 6. Differential Expression of GBMO-30 and NSCO Transcriptomes, Related to Figure 4.

SUPPLEMENTARY TABLE 7. Differential Expression of GBMO-640 and NSCO Transcriptomes, Related to Figure 2.

SUPPLEMENTARY TABLE 8. Differential Expression of GBMO-965 and NSCO Transcriptomes, Related to Figure 2.

SUPPLEMENTARY TABLE 9. Differential Expression of GBMO-1201 and NSCO Transcriptomes, Related to Figure 2.

SUPPLEMENTARY TABLE 10. Spatial Tumor Expression of GBMO-30 Upregulated Genes, Related to Supplemental Figure 6.

SUPPLEMENTARY TABLE 11. Spatial Tumor Expression of GBMO-640 Upregulated Genes, Related to Supplemental Figure 6.

SUPPLEMENTARY TABLE 12. Spatial Tumor Expression of GBMO-965 Upregulated Genes, Related to Supplemental Figure 6.

SUPPLEMENTARY TABLE 13. Spatial Tumor Expression of GBMO-1201 Upregulated Genes, Related to Supplemental Figure 6.

SUPPLEMENTARY TABLE 14. Primer Sequences for q-PCR

SUPPLEMENTARY TABLE 15. Comparison of Glioblastoma organoids
